# Photobiomodulation of 450 nm Blue Light on Human Keratinocytes, Fibroblasts, and Endothelial Cells: An In Vitro and Transcriptomic Study on Cells Involved in Wound Healing and Angiogenesis

**DOI:** 10.3390/biomedicines13081876

**Published:** 2025-08-01

**Authors:** Jingbo Shao, Sophie Clément, Christoph Reissfelder, Patrick Téoule, Norbert Gretz, Feng Guo, Sabina Hajizada, Stefanie Uhlig, Katharina Mößinger, Carolina de la Torre, Carsten Sticht, Vugar Yagublu, Michael Keese

**Affiliations:** 1 Department of Surgery, Medical Faculty Mannheim, Heidelberg University, 68167 Mannheim, Germany; jingbo.shao@medma.uni-heidelberg.de (J.S.); christoph.reissfelder@medma.uni-heidelberg.de (C.R.); sabina.hajizada@medma.uni-heidelberg.de (S.H.); 2 Urgo Research Innovation and Development, 21300 Chenôve, France; s.clement@fr.urgo.com; 3 Department of General and Visceral Surgery, Ulm University Hospital, 89081 Ulm, Germany; patrick.teoule@uniklinik-ulm.de; 4 Medical Research Centre, Medical Faculty Mannheim, Heidelberg University, 68167 Mannheim, Germany; norbert.gretz@medma.uni-heidelberg.de; 5 Department of Vascular Surgery, The First Affiliated Hospital of Chongqing Medical University, No.1 Youyi Road, Yuzhong District, Chongqing 400016, China; guofengsc12@gmail.com; 6 FlowCore Mannheim, Medical Faculty Mannheim, Heidelberg University, 68167 Mannheim, Germany; stefanie.uhlig@medma.uni-heidelberg.de; 7 Institute of Transfusion Medicine and Immunology, Franz-Volhard-Straße 6, 68167 Mannheim, Germany; 8 NGS Core Facility, Medical Faculty Mannheim, Heidelberg University, 68167 Mannheim, Germany; katharina.moessinger@medma.uni-heidelberg.de (K.M.); carolina.delatorre@medma.uni-heidelberg.de (C.d.l.T.); carsten.sticht@medma.uni-heidelberg.de (C.S.); 9 Department of Vascular Surgery, Theresienkrankenhaus, 68165 Mannheim, Germany

**Keywords:** keratinocytes, fibroblasts, endothelial cells, wound healing, angiogenesis, photobiomodulation

## Abstract

**Background**: Blue light (BL) irradiation has been shown to induce photobiomodulation (PBM) in cells. Here, we investigate its influence on cell types involved in wound healing. **Methods**: Cellular responses of immortalized human keratinocytes (HaCaTs), normal human dermal fibroblasts (NHDFs), and human umbilical vein endothelial cells (HUVECs) after light treatment at 450 nm were analyzed by kinetic assays on cell viability, proliferation, ATP quantification, migration assay, and apoptosis assay. Gene expression was evaluated by transcriptome analysis. **Results**: A biphasic effect was observed on HaCaTs, NHDFs, and HUVECs. Low-fluence (4.5 J/cm^2^) irradiation stimulated cell viability, proliferation, and migration. mRNA sequencing indicated involvement of transforming growth factor beta (TGF-β), ErbB, and vascular endothelial growth factor (VEGF) pathways. High-fluence (18 J/cm^2^) irradiation inhibited these cellular activities by downregulating DNA replication, the cell cycle, and mismatch repair pathways. **Conclusions**: HaCaTs, NHDFs, and HUVECs exhibited a dose-dependent pattern after BL irradiation. These findings broaden the view of PBM following BL irradiation of these three cell types, thereby promoting their potential application in wound healing and angiogenesis. Our data on low-fluence BL at 450 nm indicates clinical potential for a novel modality in wound therapy.

## 1. Introduction

Chronic wounds not only impact the quality of life, but they also cause a tremendous economic burden [1]. In 2019, with a 13% increase in wound incidence over a 5-year period, the expenditure on chronic nonhealing wounds in the United States reached 22.5 billion USD [2]. Moreover, considering the aging population and the continuously growing obese and diabetic population, chronic wounds are anticipated to remain a significant clinical and economic challenge [3,4].

Since Endre Mester first introduced the concept of PBM in 1967, numerous studies have focused on the effectiveness of red/near-infrared (NIR) light in wound healing [5]. For instance, Schmidt et al. conducted an in vivo study on rats, and Schmidt et al. applied 2.7 J/cm^2^ light therapy at a wavelength of 660 nm on skin wounds, which resulted in a faster rate of wound closure ten days post-exposure [6]. More recently, Tri et al. utilized merged light sources with wavelengths of 630 and 850 nm on skin wounds in diabetic mice, suggesting a positive role of light treatment after 2 J/cm^2^ irradiation [7]. Barolet et al. reported favorable outcomes in patients’ post-traumatic ulcers and hematomas using 4 J/cm^2^ pulsed irradiation at 660 nm [8]. Cytochrome c oxidase (CCO), the most widely recognized photoreceptor of visible red/NIR light, is known to stimulate electron transport chains at the mitochondrial membrane upon irradiation [9]. This activation subsequently influences intracellular signaling pathways involving nitric oxide (NO), reactive oxygen species (ROS), and adenosine triphosphate (ATP), which alter cellular metabolism, proliferation, migration, and apoptosis [10,11].

Relatively few studies report PBM on wound healing and angiogenesis after exposure to other visible light. Simões et al. demonstrated proangiogenic effects and potential myofibroblastic differentiation in skin burns of rats using an energy density of 240 J/cm^2^ irradiation at a wavelength of 520 nm [12]. Furthermore, wavelength-dependent effects have been noted after pulsed light therapy, with consecutive improvements in wound healing observed in diabetic mice attributed to green light irradiation at 540 nm [13]. Flavins and flavoproteins are believed to act as photoreceptors during the absorption of visible light at 400 and 550 nm. Flavoproteins with coenzymes of flavin mononucleotide (FMN) in complex I and flavin adenine dinucleotide (FAD) in complex II were both essential to electron-transfer chains, followed by redox reactions and energy metabolism [14].

Data on the use of blue light irradiation in wound healing are insufficient. BL with wavelengths between 400 and 500 nm has been shown to benefit wound healing in patients with various wound etiologies. For example, BL with wavelengths of 400 to 430 nm at 7.2 J/cm^2^ has been reported to promote the desired outcome in lower-limb ulceration due to chronic venous insufficiency, resulting in reduced lesion depth and revitalization of perilesional skin after 12 weeks [15]. Additionally, BL irradiation has clinically promoted wound repair in patients with peripheral artery disease (PAD) through a reduction in lesions [15]. In cases of long-lasting venous ulcers, faster reepithelialization was observed [15]. Lastly, Marcheli et al. assessed reepithelialization after BL irradiation and concluded that BL positively influences the healing process of arterial ulcers [16]. Notably, visible light in the blue spectrum has been shown to act on nitrosated proteins and release NO, a well-known vasodilator and regulator of endothelial function [17]. These findings suggested a promising role for BL irradiation in clinical applications for patients with wounds.

Other photoreceptors, such as Opsins (OPNs), were also described [18]. Opsins, as G-protein-coupled receptors, have been shown to influence the early differentiation of keratinocytes following BL irradiation [19]. Buscone et al. found that 3.2 J/cm^2^ BL irradiation stimulated the proliferation of outer root sheath keratinocytes via interaction with OPN3 [20]. Sikka et al. reported a photorelaxation response following low-intensity BL irradiation in mice mediated by OPN4 [21]. Additionally, Wang et al. demonstrated that transient receptor potential (TRP) calcium channels could regulate osteoblast differentiation after light exposure at 420 and 540 nm [22]. While these findings provide strong evidence for the effectiveness of PBM after BL, the underlying mechanisms of PBM after BL irradiation are not completely understood.

In this study, we aimed to explore the effects of BL on cells that are involved in wound healing, namely HaCaTs, NHDFs, and HUVECs. Then, optical irradiation settings based on the metabolism and proliferation levels of the three cell lines were selected to investigate the levels of migration, apoptosis, and gene expression, with the aim of understanding the effect of BL irradiation on wound healing and angiogenesis.

## 2. Materials and Methods

### 2.1. Cell Lines and Cell Culture

#### 2.1.1. Immortalized Human Keratinocytes

HaCaT, an immortalized human keratinocyte cell line (spontaneously immortalized aneuploid human keratinocyte cell line), was purchased from CLS Cell Lines Service GmbH (Eppelheim, Germany). The cells were cultured in Dulbecco’s modified Eagle medium with high-glucose (DMEM GlutaMAX^TM^, [+] 4.5 g/L glucose, [−] pyruvate, Thermo Fisher Scientific, Grand Island, New York, NY, USA) supplemented with 10% fetal bovine serum (FBS, Thermo Fisher Scientific, Grand Island, New York, NY, USA), 1 mM sodium pyruvate (Sigma-Aldrich Chemie GmbH, Taufkirchen, Germany), 10,000 units penicillin, and 10 mg streptomycin per mL (pen/strep, Sigma-Aldrich Chemie GmbH, Taufkirchen, Germany). The cells were cultured at 37 °C in a humidified atmosphere with 5% CO_2_ and were passaged at 1:4 or 1:3 for use every two to three days, depending on confluence.

#### 2.1.2. Normal Human Dermal Fibroblasts

NHDFs isolated from the pooled dermis of juvenile foreskin or adult skin were purchased from PromoCell GmbH (Heidelberg, Germany). They were cultivated in DMEM GlutaMAX™ (Thermo Fisher Scientific) supplemented with 10,000 units of penicillin and 10 mg streptomycin per mL. They were also supplemented with Fibroblast Growth Medium 3 from PromoCell (Heidelberg, Germany) and 1 mM sodium pyruvate. Like keratinocytes, NHDF cultures were also maintained at 37 °C under 5% CO_2_. NHDFs were passaged at a passaging ratio of 1:4 or 1:3 every three days until passage 10.

#### 2.1.3. Human Umbilical Venous Endothelial Cells

HUVECs were isolated from human umbilical cords using the methods described by Jaffe et al. [23]. Umbilical cords were obtained from placentae soon after birth. Briefly, two pharmaceutical glass tubes were cannulated and fixed to both ends of an umbilical vein, followed by washing with DPBS (Gibco™, Grand Island, New York, NY, USA) to drain clots. After digestion with 1% collagenase IV (Sigma-Aldrich, Taufkirchen, Germany) for 10 min, the vein was flushed with endothelial growth medium (Provitro, Berlin, Germany) with 5% FBS (Gibco, Brasilia, Brazil), and the collected HUVECs were subsequently centrifuged. The umbilical cords were obtained from donors from the Gynecology and Obstetrics Department of Universitätsklinik Mannheim, and HUVEC isolation was approved by the local ethics committee (Medizinische Ethik-Kommission II, Medizinische Fakultät Mannheim, 2015-581N-MA, Mannheim, Germany, approval date: 24 February 2015). The isolated endothelial cells were maintained at 37 °C under 5% CO_2_ in endothelial cell growth medium supplemented with 5% FBS, endothelial cell growth supplement mix (Provitro, Berlin, Germany), 10,000 units of penicillin, and 10 mg streptomycin per mL. HUVEC cultures were maintained at a passaging ratio of 1:3. HUVECs were isolated from nine human umbilical cords (donors), and three donors were pooled and used in experiments between passages two and five. Morphologies of HUVECs are presented in Figure S1.

#### 2.1.4. Treatment Protocol on Cell Culture Prior to and After BL Irradiation

All adherent cell lines were refreshed with medium refreshment 24 h after cells were seeded on plates. Subsequently, cells were incubated at 37 °C with 5% CO_2_ for another 30 min, which was before BL irradiation. Specifically, the corresponding medium was renewed only before cells were exposed to BL irradiation. Both fresh medium and cells were simultaneously exposed to light doses. During the irradiation period, culture plates were kept under aseptic conditions with a sterile lid in place at all times. Culture sterility was confirmed by microscopic inspection 24 h after irradiation for all cell lines.

### 2.2. BL Irradiation Device and Photodiode and Measurement of Light Intensity

The LED panel (10 × 10 LED matrix) served as the light source (Osram Duris S5, GD PSLR31, Austria) with irradiances of 7, 10, and 23 mW/cm^2^ at an irradiation distance of 5.4 cm (Figure S2). Visible light at a wavelength of 450 ± 5 nm (peak wavelength: 447 nm, Figure S3) in continuous irradiation mode was emitted by a prototype design from URGO (URGO RID, Chenôve, France). Through the attached heatsinks beneath the reflective box, heat generated during irradiation was passively evacuated. The temperature was measured in the medium, above the medium, and between two wells (Figure S4). Additionally, a spectrophotometer (AVANTES AvaSpec-3648, Apeldoorn, The Netherlands) was used for calibration by URGO. The BL irradiation times ranged from 2.5 to 120 min. The distribution of the BL was quantified with an irradiance of 23 mW/cm^2^ using a REUR2021-085SAF photodiode-based device and a metal plate (Dibond, aluminum composite material) from URGO (Figure S5). The photodiode was attached to the bottom of each well on the metal plate, and the corresponding current value was recorded.

### 2.3. Cell Viability Assay

Cell viability was assessed using a Colorimetric Cell Viability Kit III (XTT) from PromoKine (PromoCell GmbH, Heidelberg, Germany). A total of 8 × 10^3^ cells/well were seeded in a black 96-well plate (Corning Incorporated, Lowell, MA, USA). Irradiation was initiated with predefined exposure times of 2.5 to 120 min and irradiances of 7, 10, and 23 mW/cm^2^. After 24 h of incubation at 37 °C and 5% CO_2_, an XTT assay was performed according to the manufacturer’s recommendations. The raw absorbance data were normalized to those of non-irradiated controls.

### 2.4. Cell Proliferation Assay

A colorimetric ELISA-BrdU kit (Roche Diagnostics GmbH, Mannheim, Germany) was used to quantify the number of newly synthesized cells and cell proliferation in a black 96-well plate after irradiation. BrdU labeling was performed 1 h after the predefined irradiation time on the second day. After incubation for 24 h, cell proliferation assays were performed according to the manufacturer’s instructions. For quantification, spectrophotometric absorption measurements were performed at 450 nm (with a reference wavelength of 690 nm). The raw data were normalized to the data from controls on the same plate.

### 2.5. ATP Quantification

A CellTiter-Glo^®^ Luminescent Cell Viability Kit (Promega Corporation, Madison, WI, USA) was used for ATP quantification. In accordance with the protocol from the manufacturer, 24 h after irradiation, the same volume (100 μL/well) of reagent as that used for the cell culture medium was added to a black 96-well plate. The mixture was subsequently shaken for 2 min to promote cell lysis. The luminescent signal was stabilized at room temperature for 10 min before the luminescence was recorded. The raw data were normalized to controls.

### 2.6. Migration Assay

A total of 8 × 10^4^ cells/well were seeded on a 12-well plate and incubated until they reached confluence. A scratch was made with a 10 μL sterile pipette tip. Before irradiation, the detached cells were washed with DPBS twice, and 1% FBS fresh cell culture medium was added. Images were captured with an inverted microscope (Carl Zeiss Microscopy, Göttingen, Germany) at predefined time intervals (0, 3, 6, 12, 24, 36, and 48 h) until the gap completely closed. The images were analyzed using ImageJ software (version 1.53e, NIH Image, NIH, Bethesda, MD, USA). The equation for wound closure was established as follows: wound closure (%) = (original wound area − area at each time point)/original wound area.

### 2.7. Apoptosis Assay

The cells were seeded at a density of 1.6 × 10^5^ cells/well in a 6-well plate. In accordance with the predefined group design, in the positive control group, the cells were treated with 1 μM staurosporine (Sigma-Aldrich, Darmstadt, Germany) 24 h prior to harvesting. The cells in the other treatment groups were exposed to different doses of BL. The cells in the control group received no intervention and were harvested simultaneously. In accordance with the manufacturer’s instructions, after trypsinization, all viable, apoptotic, and dead cells in the supernatants were completely harvested and centrifuged. Then, 0.5 μL of Annexin V and 2.5 μL of propidium iodide (PI) (FITC Annexin V Apoptosis Detection Kit I, BD Pharmingen^TM^, Franklin Lake, NJ, USA) were added after titration. Moreover, unstained cells were harvested. Then, the mixture was vortexed and incubated in the dark at room temperature for 15 min. Finally, after adding 1X binding buffer, a maximum of 10,000 events/tube were recorded with a BD FACSCanto II (BD Bioscience, Heidelberg, Germany) and analyzed with FlowJo v10 (FlowJo LLC, Ashland, OR, USA).

### 2.8. RNA Isolation and Sequencing

Total RNA was isolated with an RNeasy Mini Kit (Qiagen, Hilden, Germany) according to the manufacturer’s instructions. The quantification of total RNA samples was performed using a NanoQuantPlate and a Spark microplate reader (Tecan, Männedorf, Switzerland). The quality check was completed with an Agilent 2100 bioanalyzer. Samples with purities (A260/280) ranging from 1.8 to 2.2 and RNA integrity numbers (RINs) above 7 were selected. The samples of total RNA were delivered to the NGS Core Facility (Mannheim, Germany) for further sequencing. The library preparation was performed using the Stranded mRNA Prep Ligation kit and sequenced on a NextSeq 550, both from Illumina (Illumina, Inc., San Diego, CA, USA).

#### 2.8.1. Bioinformatic Analysis

RNA-seq data processing was performed with R (version 4.3.0) and a bioconductor (version 3.19) in RStudio (version 1.1.463). Quality control of the clean sequencing reads was performed using FastQC (Babraham Bioinformatics, Cambridge, UK). Low-quality reads were removed using trim_galore (version 0.6.4). The resulting reads were aligned to the human genome version GRCh38.p13 and counted using kallisto version 0.46.1 [24]. The count data were transformed to log_2_-counts per million (logCPM) values using the voom function in the limma package (3.61.3) [25]. Differential expression analysis was performed using the limma package in R. A false-positive rate of α = 0.05 with false discovery rate (FDR) correction was used as the level of significance. Volcano plots and heatmaps were created using the ggplot2 package (version 3.5.0) and the complexHeatmap package (version 2.0.0) [26]. For enrichment analysis, the fgsea [27], enrichmentbrowser [28], and Enrichr packages [29] were used. Pathway analysis was performed with the fgsea and enrichmentbrowser packages in R using pathway information from the KEGG database (“https://www.genome.jp/kegg/pathway.html (accessed on 12 August 2024)”). The protein–protein interaction (PPI) network was constructed in Cytoscape (3.10.2) after the differentially expressed genes were retrieved from Search Tool for the Retrieval of Interacting Genes (STRING), which was accessed on 17 August 2024. CytoHubba was used to explore the importance of nodes in networks constructed from differentially expressed genes.

#### 2.8.2. Statistical Analysis

The data were analyzed using GraphPad Prism (version 8.0.2, San Diego, CA, USA) with a paired Student’s t-test or repeated measures ANOVA (RM AVOVA) to compare groups. The Shapiro–Wilk normality test was selected for testing normality. In RM ANOVA, Greenhouse–Geisser correction was applied if Mauchly’s test *p*-value < 0.05. All numerical data are expressed as either the mean ± standard error of the mean (SEM) or median, interquartile range (25th and 75th percentiles, IQR), maximum, or minimum. A *p*-value < 0.05 was considered statistically significant.

## 3. Results

### 3.1. Distribution of Light Intensity

Before the in vitro experiments on the cells, we investigated the light homogeneity in the 96-well plate. The values of light intensity were normalized to the maximum value, which was measured using a photodiode-based device specific for the 450 nm BL device, when the irradiance reached 23 mW/cm^2^. The light intensities in the wells at the plate edge were 10% lower than those measured in the center (Figure S6). As a result, those wells on the edge of the plate were not used for the in vitro experiments. 

### 3.2. Effects of BL Irradiation on Cell Viability and Proliferation at Different Irradiances

A clear biphasic response to BL irradiation was observed in all the cell lines at all the irradiances (7, 10, and 23 mW/cm^2^) used in the XTT assay after converting the parameters (irradiation time and irradiance) into fluence levels (Table S1).

**Figure 1:**
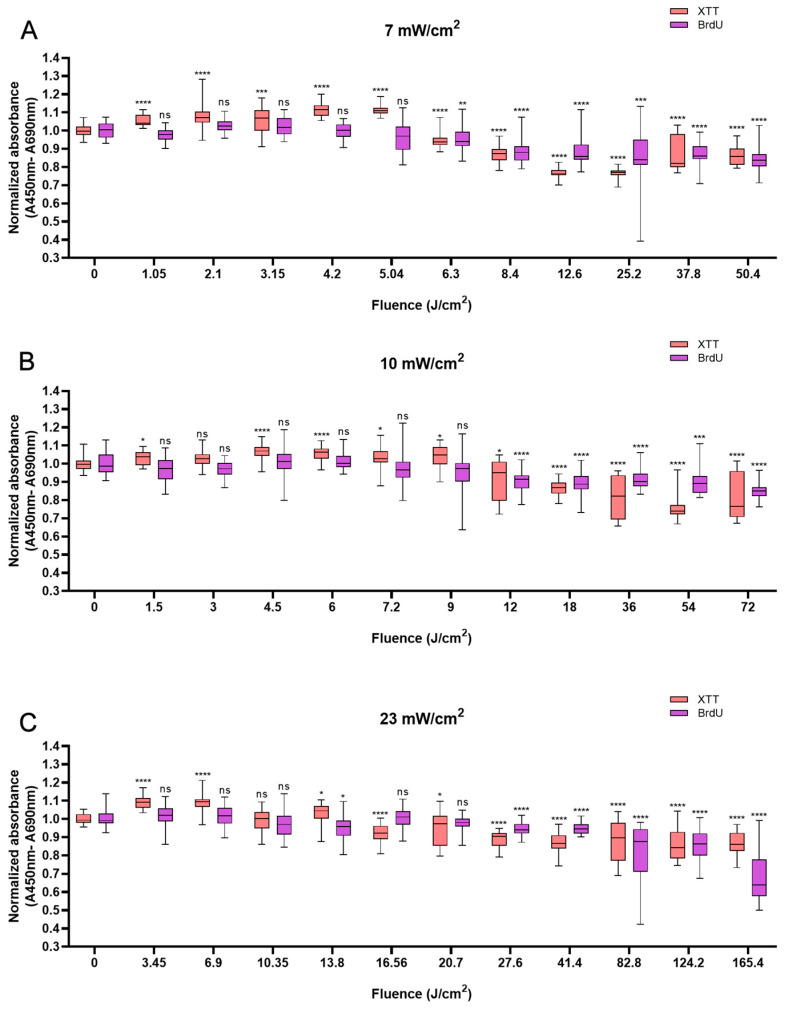
Response of HaCaTs in cell viability and proliferation after BL irradiation with 0–120 min of irradiation at three irradiance levels: 7, 10, and 23 mW/cm^2^ (**A**–**C**). For both assays, the absorbance was normalized and compared to that of non-irradiated controls. The data are shown as boxplots with medians, IQRs, and error bars for outliers (n = 3 repetitions, *: *p* < 0.05, **: *p* < 0.01, ***: *p* < 0.001, ****: *p* < 0.0001, ns: not significant; RM ANOVA: Dunnett’s test).

In HaCaTs, BL irradiation at a fluence of 5.04 J/cm^2^ with an irradiation time of 12 min triggered a maximum increase in XTT absorbance of 11.5% compared with that in non-irradiated cells (Figure 1A). BL irradiation at the energy density of 4.5 J/cm^2^ for 7.5 min resulted in a 6.7% increase (Figure 1B), and with the fluence of 3.45 J/cm^2^, the cellular activity increased by 9.5% at 2.5 min (Figure 1C). After 20 min of irradiation at all irradiances, the cell viability decreased and was significantly lower than that of the control group (*p* < 0.05). With 120 min of irradiation irrespective of irradiance, a reduction in cell viability was observed with 13.8% for 50.4 J/cm^2^, 19.8% for 72 J/cm^2^, and 13.5% for 165.6 J/cm^2^, respectively (*p* < 0.0001), in comparison to non-irradiated cells (Figure 1A–C).

Not all changes in cell viability were accompanied by corresponding changes in cellular proliferation. However, a clear decrease in cellular proliferation was observed after irradiation for longer than 20 min. A 15.9% decrease in cell proliferation was observed with 120 min of irradiation at 50.4 J/cm^2^ (*p* < 0.0001, Figure 1A), a 15.1% decrease was observed with irradiation at the fluence of 72 J/cm^2^ for 120 min (*p* < 0.0001, Figure 1B), and a 31.2% decrease was observed with irradiation for 120 min (165.6 J/cm^2^, *p* < 0.0001), in comparison with the control (Figure 1C).

**Figure 2:**
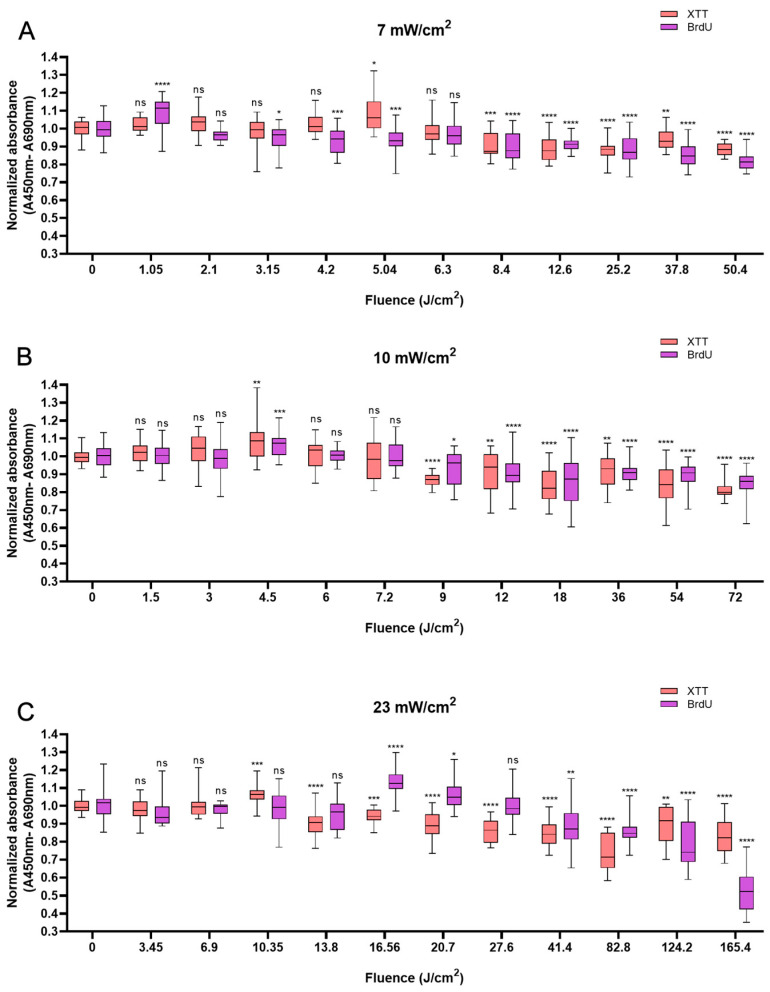
Overview of the response of NHDFs to BL with 0–120 min of irradiation by XTT and BrdU assays at 7, 10, and 23 mW/cm^2^. The absorbance was normalized and compared to that of non-irradiated controls. (**A**): Changes in cell viability and cell proliferation under the light irradiance of 7 mW/cm^2^ 24 h after BL irradiation, with fluence ranging from 0 to 50.4 J/cm^2^. (**B**): Normalized absorbance from XTT and BrdU 24 h after irradiation and grouped by fluence from 0 to 72 J/cm^2^, as shown on the x-axis. (**C**): Cell viability and proliferation responses of NHDFs after 23 mW/cm^2^ irradiation, with fluence ranging from 0 to 165.4 J/cm^2^. The data are shown as boxplots with medians, IQRs, and error bars for outliers (n = 3 repetitions, *: *p* < 0.05, **: *p* < 0.01, ***: *p* < 0.001, ****: *p* < 0.0001, ns: not significant; RM ANOVA: Dunnett’s test).

Like HaCaTs, NHDFs also responded in a biphasic manner to BL irradiation, with an increase in cell viability at a shorter irradiation duration of up to 12 min and a decrease at a longer irradiation duration, i.e., longer than 20 min. Whereas a modest increase of 10% was observed with 5.04 J/cm^2^ at 12 min (Figure 2A), a peak value of 8.9% higher than that of the control group was observed with irradiation at the fluence of 4.5 J/cm^2^ at 7.5 min (Figure 2B). An increase of 6.4% was noted when 10.35 J/cm^2^ BL was used at 7.5 min (Figure 2C). Cellular proliferation also exhibited biphasic characteristics. When light treatment with 1.05 J/cm^2^ BL irradiation was applied, cell proliferation increased by 8.8% at 2.5 min. Paralleling an increase in fluence from 4.5 to 16.56 J/cm^2^, a minor increase in cellular proliferation was observed from 6.8% at 7.5 min (*p* < 0.001) to 13.2% at 12 min (*p* < 0.0001; Figure 2B,C). Compared with those of the control cells, the proliferation of the cells exposed to BL irradiation for longer than 20 min decreased, and with 165.6 J/cm^2^ BL irradiation, the cell proliferation decreased by 47.8% (*p* < 0.0001, Figure 2C). With 50.4 and 72 J/cm^2^ BL irradiation, a decrease in cellular proliferation of 18.1% and 15.1%, respectively, was also observed in Figure 2B,C, yet the magnitude was much lower than that with 23 mW/cm^2^ BL irradiation.

**Figure 3:**
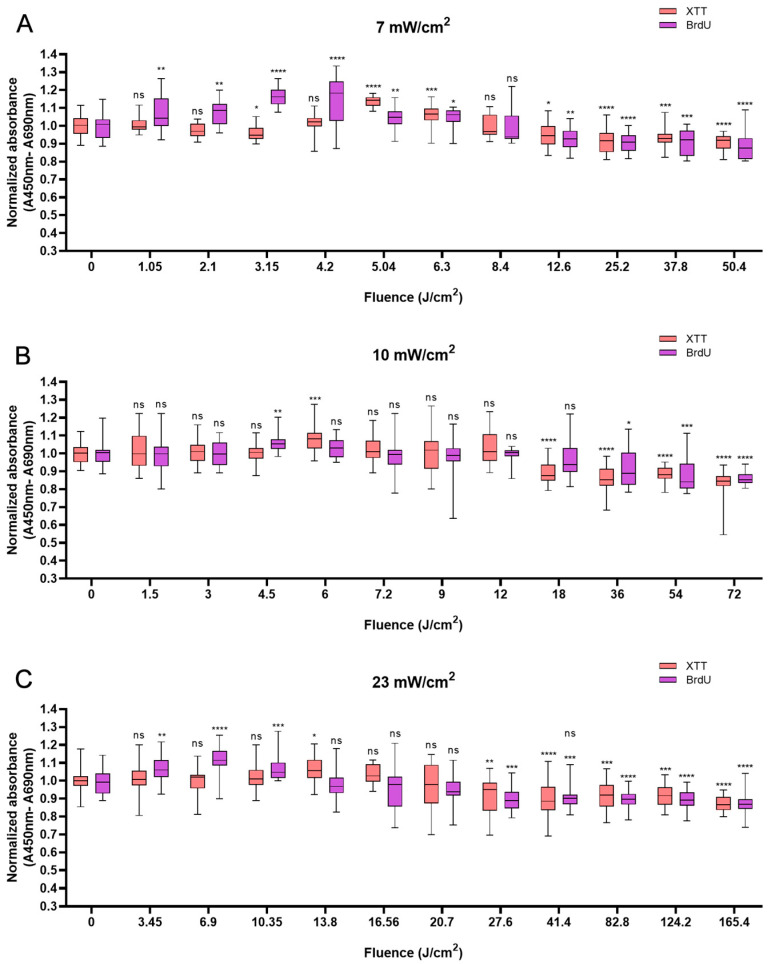
Overview of the response of HUVECs to BL over 0–120 min with 7, 10, and 23 mW/cm^2^ by XTT and BrdU assays. The absorbance was normalized and compared to that of non-irradiated controls. (**A**): The normalized absorbance measured 24 h after BL irradiation (7 mW/cm^2^); the duration of light treatment varied from 0 to 120 min. (**B**,**C**): Changes in newly synthesized DNA and cell viability 24 h after BL irradiation with irradiances of 10 and 23 mW/cm^2^. The data are shown as boxplots with medians, IQRs, and error bars for outliers (n = 3 repetitions, *: *p* < 0.05, **: *p* < 0.01, ***: *p* < 0.001, ****: *p* < 0.0001, ns: not significant; RM ANOVA: Dunnett’s test).

HUVECs also responded biphasically to BL irradiation. Compared with the non-irradiated control, irradiation at an energy density of 5.04 J/cm^2^ caused a peak in cell viability of 13.7% at 12 min (Figure 3A). At 10 min, higher power densities (10 and 23 mW/cm^2^) led to peaks of 8.1% and 5.9% at fluences of 6 J/cm^2^ and 13.8 J/cm^2^, respectively (Figure 3B,C). Like in HaCaTs and NHDFs, an increase in cell viability did not correspond to an increase in cell proliferation in HUVECs. Irradiation with energy densities of 3.15, 4.5, and 6.9 J/cm^2^ increased proliferation by 16.1% (*p* < 0.0001) at 7.5 min, 5.9% (*p* < 0.01) at 7.5 min, and 11.7% (*p* < 0.0001) at 5 min, respectively. With an irradiation time of longer than 20 min, a decrease in cell proliferation was observed, with decreases of 11.3% and 14.0% compared with those of the controls obtained with 50.4 J/cm^2^ and 72 J/cm^2^ BL irradiation at 120 min, respectively. Compared with the non-irradiated group, the 165.6 J/cm^2^ BL irradiation induced a slight decrease of 12.6% (*p* < 0.0001) in cell proliferation at 120 min (Figure 3C).

In this in vitro study, we focused on the biostimulation effect of BL irradiation on three cell types, which prominently exert biological effects on wound healing and angiogenesis. For the subsequent ATP quantification experiments, we chose an irradiance of 10 mW/cm^2^ for durations of 7.5 and 10 min based on the responses of HaCaTs, NHDFs, and HUVECs to BL.

### 3.3. Effects of BL on ATP Quantification

**Figure 4:**
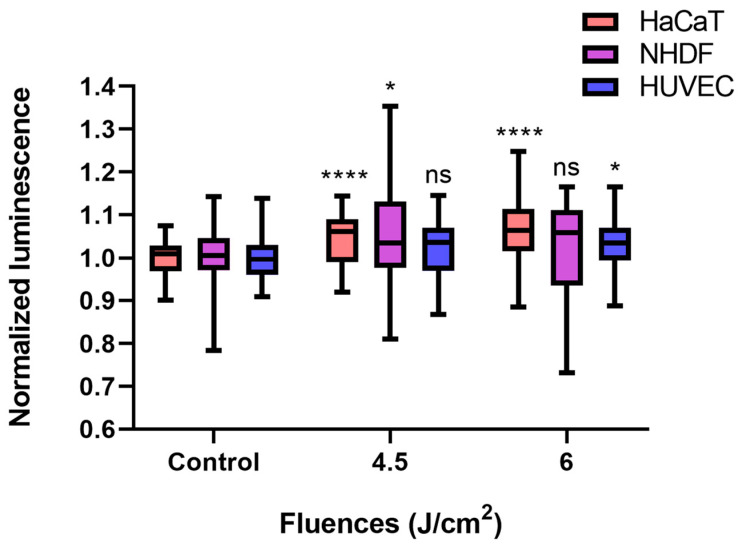
BL irradiation altered ATP quantification in HaCaTs, NHDFs, and HUVECs at different doses (control, 4.5, and 6 J/cm^2^). The data are shown as boxplots with medians, IQRs, and error bars for outliers (n = 3 repetitions, *: *p* < 0.05, ****: *p* < 0.0001, ns: not significant, RM ANOVA: Dunnett’s test). The luminescence was normalized to that of the controls without irradiation.

Compared with the control conditions, low-fluence (4.5 J/cm^2^, 7.5 min × 10 mW/cm^2^) BL irradiation increased ATP levels in each cell type, even though only a 2.2% increase was detected in HUVECs (*p* > 0.05). In terms of the NHDF, the ATP amount significantly increased by 5.4% (*p* < 0.05), whereas a fluence of 6 J/cm^2^ (10 min × 10 mW/cm^2^) did not significantly alter the ATP concentration of the NHDFs. The normalized luminescence levels of HaCaTs and HUVECs, which were proportional to the ATP amount, increased over time and reached 1.064 (*p* < 0.0001) and 1.033 (*p* < 0.05) after 6 J/cm^2^ irradiation, respectively (Figure 4).

Therefore, a fluence of 4.5 J/cm^2^ BL was used for further studies on cell migration, apoptosis, and gene expression.

### 3.4. Effects of 4.5 J/cm^2^ BL on Cell Migration

To assess whether light treatment has the ability to stimulate cellular migration, scratch assays were designed after a fluence of 4.5 J/cm^2^ BL was applied.

**Figure 5:**
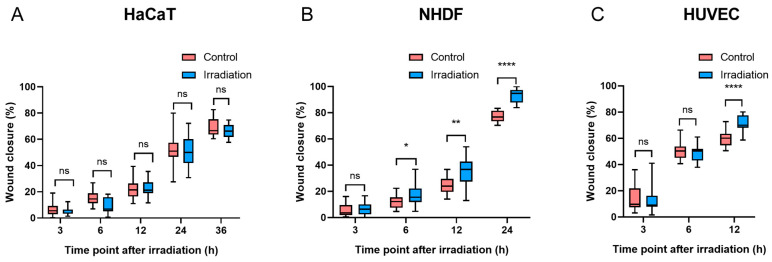
Migration was quantified at different time points after BL in three cell types ((**A**): HaCaTs, (**B**): NHDFs, and (**C**): HUVECs). The data are shown as boxplots with medians, IQRs, and error bars for outliers (n = 3 repetitions, *: *p* < 0.05, **: *p* < 0.01, ****: *p* < 0.0001, ns: not significant, paired Student’s *t* test).

Compared with the controls, there was no significant difference in wound closure in HaCaT cells at any time point after irradiation (*p* > 0.05, Figure 5A). Six hours after irradiation, NHDFs exposed to 4.5 J/cm^2^ had a significantly faster wound closure rate than did the controls (*p* < 0.05). Furthermore, as NHDF migration progressed, the difference in wound closure increased after another 6 h (10.7% vs. 5.8%) (Figure 5B), and such progressive divergence between BL-treated NHDFs and controls became more pronounced at 24 h, with wound closure reaching 77.3% in controls and 93.2% in the irradiation groups. In terms of HUVEC migration, a significant difference was observed only at 12 h after irradiation (*p* < 0.0001) (Figure 5C). Both NHDFs and HUVECs experienced complete or near-complete wound closure within 24 h after irradiation, whereas HaCaTs demonstrated slower wound closure percentages than did NHDFs and HUVECs, with values greater than 65% in both the control and irradiation groups at 36 h (Figure 5A), which was longer than the observation time required for wound closure in NHDFs and HUVECs. The representative images of wound closure were captured at corresponding times after irradiation (Figure S7: HaCaTs, Figure S8: NHDFs, and Figure S9: HUVECs).

### 3.5. Effects of BL on Cell Apoptosis

**Figure 6:**
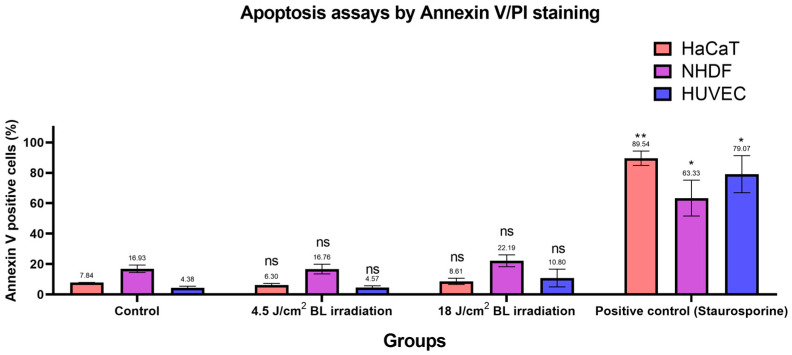
Changes in the number of apoptotic cells among the 4.5 and 18 J/cm^2^ irradiation, 1 μM staurosporine, and control groups were measured by Annexin V/PI staining. The data are shown as column bars with the means ± SEMs, with mean values indicated above error bars (n = 3 repetitions, *: *p* < 0.05, **: *p* < 0.01, ns: not significant; RM ANOVA: Dunnett’s test).

Based on the previous findings [30,31], we designed apoptosis assays with Annexin V/PI staining to evaluate apoptotic conditions after irradiation with 4.5 and 18 J/cm^2^ (30 min × 10 mW/cm^2^). Annexin V+/PI− and Annexin V+/PI+ cells were considered apoptotic. As shown in Figure 6, apoptosis in the irradiation groups (4.5 and 18 J/cm^2^ BL) was not significantly different from the levels in the corresponding control groups among HaCaTs, NHDFs, and HUVECs. After sorting, only cells exposed to 1 μM staurosporine for 24 h in the positive control group presented significantly increased apoptotic levels, as measured by flow cytometry. Exemplary results of Annexin V/PI staining on HaCaTs, NHDFs, and HUVECs grouped by control, 4.5, 18 J/cm^2^ BL irradiation, and positive control are shown in Figure S10. Accordingly, the proportions of cells in each quadrant were compared between treatment groups and controls in Figure S11.

### 3.6. RNA Sequencing and Gene Expression Analysis

**Figure 7:**
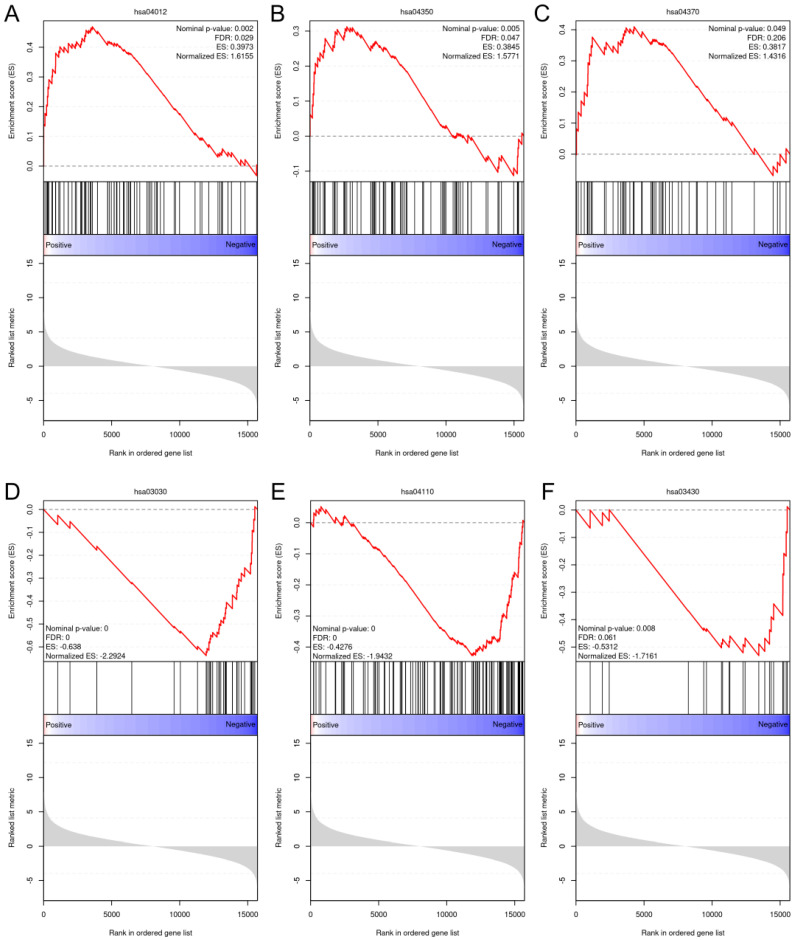
GSEA plots of the upregulated and downregulated gene sets enriched in different pathways from the KEGG database. (**A**): ErbB signaling pathway (hsa04012); (**B**): TGF-β signaling pathway (hsa04350); (**C**): VEGF signaling pathway (hsa04370); (**D**): DNA replication pathway (hsa03030); (**E**): cell cycle (hsa04110); and (**F**): mismatch repair pathway (hsa03430).

After an overall assessment of cellular responses after various doses of BL, two fluences, 4.5 and 18 J/cm^2^, were selected to explore the impacts of light treatments on gene expression. Because NHDFs play a profound role in signaling cascades and interact with keratinocytes and endothelial cells throughout the phases of wound healing and angiogenesis, we decided to perform RNA sequencing using NHDFs. The correlation heatmap (Figure S12) revealed good reliability and repeatability, with distinct clusters. After differential expression analysis, 291 out of the 621 genes were upregulated compared with the controls after exposure to 4.5 J/cm^2^. The expression levels of genes known to regulate cellular viability, motility, and proliferation, such as MET, EREG, and MEMO1, were significantly increased. Two collagen genes, COL5A3 and COL8A1, were also significantly upregulated. When the light dose increased to 18 J/cm^2^, 1376 genes were upregulated, which was greater than the 1265 genes whose expression levels were downregulated in the 18 J/cm^2^ group (Figure S13). After gene set enrichment analysis (GSEA), only pathways from the KEGG database that met the following thresholds were considered significantly enriched: FDR < 0.25, nominal p value < 0.05, and normalized enrichment score (NES) > 1 or NES < −1. The ErbB pathway (NES = 1.62), the TGF-β pathway (NES = 1.58), and the VEGF pathway (NES = 1.43) were enriched after 4.5 J/cm^2^ irradiation (Figure 7A–C). After 18 J/cm^2^ irradiation, the downregulated genes were enriched in DNA replication (NES = −2.29), the cell cycle (NES = −1.94), and mismatch repair (NES = −1.72) (Figure 7D–F).

**Figure 8:**
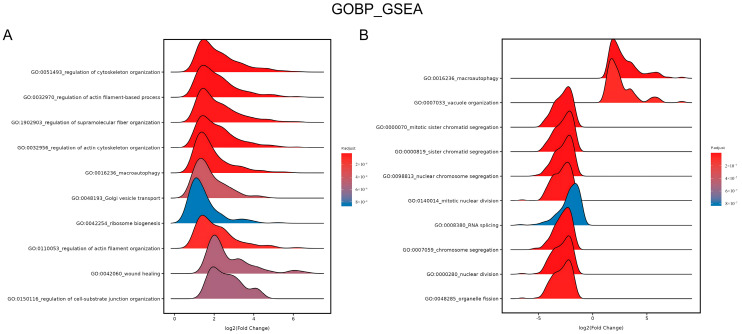
Ridge plots of enrichment in GO BP after 4.5 J/cm^2^ (**A**) and 18 J/cm^2^ (**B**) BL on NHDFs. The plots show the enriched GO terms, *p* values, and log_2_ (fold change) values.

Furthermore, gene ontology (GO) term enrichment was applied. The ridge plot in Figure 8A shows the enrichment in the GO biological process (BP) category after GSEA. The upregulated genes were significantly enriched in wound healing (GO:0042060) after 4.5 J/cm^2^ BL irradiation. Figure 8B shows that the downregulated genes were enriched in several processes of mitosis, including mitotic sister chromatid segregation (GO:0000070), chromosome segregation (GO:0007059), and nuclear division (GO:0000280), after 18 J/cm^2^ irradiation (*p*.adjust < 0.0001).

Moreover, additional GSEA was conducted using the Reactome database to provide more information on pathway enrichment. Figure S14A shows that 4.5 J/cm^2^ BL irradiation upregulated genes involved in translation, whereas 18 J/cm^2^ BL downregulated genes involved in the mRNA splicing pathway (Figure S14B).

**Figure 9:**
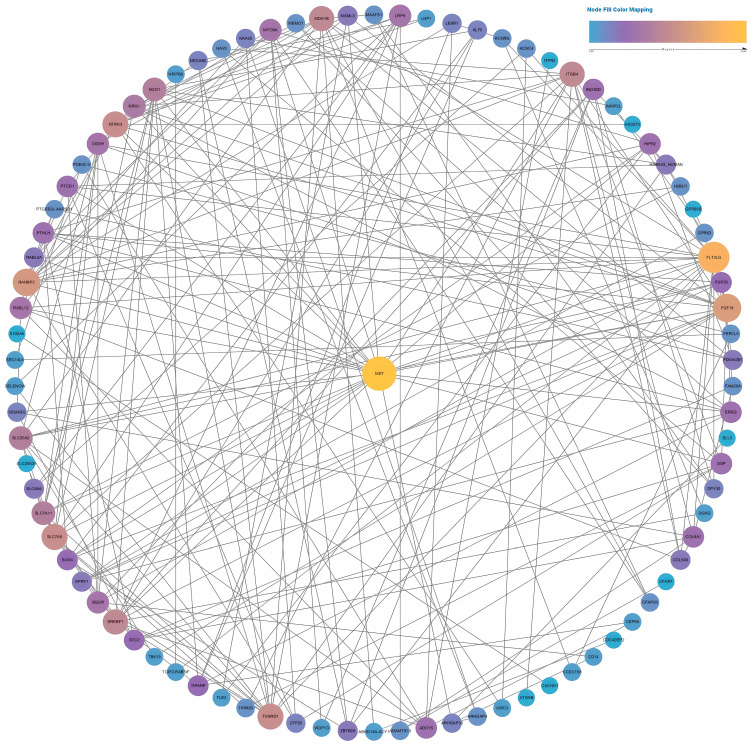
Full PPI network of the differentially expressed genes in the low-fluence irradiation group. The size and color of the nodes indicate the node degree.

Two PPI networks were constructed based on data from the STRING database. We selected an interaction score >0.15 to construct a network for the low-fluence (4.5 J/cm^2^) group and an interaction score >0.7 for the high-fluence (18 J/cm^2^) group. As shown in Figure 9, the PPI network contained 87 nodes of differentially expressed genes. MET achieved the highest degree, ranking No. 1 by the maximal clique centrality (MCC) method in CytoHubba. After calculation, MET had a much higher score than the second gene, TXNRD1 (157 in MET vs. 91 in TXNRD1). As a result, MET was identified as a hub gene containing 24 connections with other genes in the network.

In terms of high-fluence irradiation on NHDFs, there were up to 498 nodes in the network (Figure S15). Among those differentially expressed genes, CDC20 ranked first, followed by AURKB, CCNB1, CCNB2, PLK1, CDCA3, TPX2, PTTG1, and SPAG5, after calculation. All these genes had scores exceeding 41,000, indicating that they were hub genes in the high-fluence group. TP53 presented the highest node degree of 64, which indicated broad connections between TP53 and other differentially expressed genes after 18 J/cm^2^ irradiation.

## 4. Discussion

In our in vitro study on BL irradiation, we observed the dose-dependent effects on the cellular behaviors of HaCaTs, NHDFs, and HUVECs through assays of viability and proliferation. A low fluence ranging from 1.05 to 16.56 J/cm^2^ can stimulate metabolic or proliferative levels. Whereas, the increasing radiant exposure gradually triggered inhibitory biological effects, irrespective of the cell type. After BL exposure for 30 min, there were clear trends of decrease in cell viability and proliferation across all irradiances. The cell type-specific responses after BL were determined after evaluating the viability, proliferation, and migration of the three cell types at different time points. Apoptosis assays revealed that neither 4.5 nor 18 J/cm^2^ irradiation caused increased levels of apoptosis and necrosis. Finally, the mechanism of the biphasic effect was revealed by RNA sequencing: 4.5 J/cm^2^ BL predominantly activated the ErbB, TGF-β, and VEGF pathways. In contrast, 18 J/cm^2^ mainly inhibited DNA replication, the cell cycle, and mismatch repair.

Dose-dependent biphasic effects have been increasingly reported in in vitro and in vivo studies since the first introduction of the concept of the “Arndt–Schulz law” in 1887 [32]. In terms of visible light in the blue spectrum, the antimicrobial, antiviral, and anti-inflammatory properties of BL (wavelengths ranging from 400 to 500 nm) have been widely reported [33,34,35]. Owing to the susceptibility of strains to relatively high-fluence BL, this treatment is a potential alternative therapy to antibiotic therapies. Furthermore, the discovery of biphasic manners of cells after BL underscores potential roles of BL in wound healing [19,33,36,37]. Masson-Meyers et al. conducted an in vitro study in which 3, 5, 10, and 55 J/cm^2^ were applied to human dermal fibroblasts, which demonstrated the biphasic effects of fibroblasts on migration and protein synthesis. These authors also demonstrated the anti-inflammatory effects of increasing doses of BL [33]. Mignon et al. used low (2 J/cm^2^) and high (30 J/cm^2^) radiant exposure to papillary and reticular fibroblasts and reported that low doses stimulated metabolic activities, whereas high doses inhibited papillary fibroblasts [36]. Similarly, Castellano-Pellicena et al. suggested keratinocyte dose-dependent responses. The levels of migration and metabolism after 2 J/cm^2^ BL irradiation were greater than those after 30 J/cm^2^ irradiation [19]. These findings suggest that low-dose irradiation promotes metabolism, migration, and function in cells. Our studies also revealed that the cell lines responded biphasically to different irradiation doses. Moreover, with more assays and irradiation times (doses), clear trends of viability and proliferation were observed after BL irradiation in HaCaTs, NHDFs, and HUVECs. BL irradiation (450 nm) at the low fluence of 4.5 J/cm^2^ clearly exhibited stimulatory effects on the above-mentioned cell lines in our in vitro study, whereas 18 J/cm^2^ irradiation affected all the cells in an inhibitory manner. Furthermore, comprehensive assessments using ATP quantification and migration assays confirmed that BL promoted ATP synthesis and migration (except for HaCaTs). The results of additional apoptosis assays demonstrated that light doses up to 18 J/cm^2^ did not induce apoptosis or necrosis in cells. This is important in terms of a potential therapeutic use of BL irradiation in wound therapy.

In addition, a gradual decline in cell viability and proliferation after intermediate radiant exposure between 4.5 and 18 J/cm^2^ clearly indicated all cell lines experienced transitions from biostimulation to the inhibitory phase. This pattern further supports the existence of biphasic cellular responses after BL irradiation, and justifies 4.5 and 18 J/cm^2^ as representative benchmarker doses throughout our study.

Notably, cell-type-specific responses after BL treatment were also observed in other in vitro studies. Rossi et al. generated a biphasic dose curve for proliferation and metabolism after 24 and 48 h of irradiation. The metabolic response of HaCaT cells was not parallel to that of fibroblasts after low-fluence irradiation (3.43 J/cm^2^) [38]. Teuschel et al. compared the levels of proliferation, migration, apoptosis, and necrosis among fibroblastic, myoblastic, and keratinocytic cell lines after treatment with 30 J/cm^2^ for 5 consecutive days and reported differences in proliferation levels and necrosis rates across cell types [39]. Mignon et al. reported striking findings of opposite responses when they added identical irradiation settings (30 J/cm^2^) to papillary and reticular fibroblasts [36]. Magni et al. utilized BL irradiation at wavelengths ranging from 410 to 430 nm and witnessed diverse cellular responses of different tissue-derived fibroblasts in terms of metabolism and proliferation. Healthy fibroblasts were more susceptible to low-fluence BL irradiation than keloid-derived fibroblasts and perilesional keloid fibroblasts in terms of cell metabolism. Conversely, these healthy fibroblasts were resistant to high-fluence BL irradiation on cell viability [40]. The existence of cell-type-dependent responses was also noted when comparing primary keratinocytes and HaCaTs after similar light treatments. Rossi et al. reported that the proliferation of HaCaTs, measured by a Sulforhodamine B-based assay, was not significantly affected by 3.43 J/cm^2^ BL irradiation [38], while Buscone et al. observed a significantly increased level of cell proliferation measured by 5-Ethynyl-2′-deoxyuridine (EdU) after primary human outer root sheath keratinocytes were exposed to 3.2 J/cm^2^ BL irradiation [20]. Similar doses of BL can induce markedly different cellular responses, revealing that different cell lines behave differently in response to light exposure. Our study demonstrated cell-type-specific responses of three cell lines, suggesting that NHDFs and HUVECs responded similarly to BL treatments in terms of cell viability, proliferation, and migration. However, keratinocytes were not susceptible to the effects of low-fluence BL irradiation on proliferation and migration. Quent et al. reported the existence of non-linear changes and miscorrelation between cellular metabolism and DNA content, particularly noting that the level of metabolism varies throughout the cell cycle [41]. An in vitro study conducted by Balzer et al. also revealed an indirect correlation between cell activity and Hoechst-stained nuclei counts [42]. Similarly, after comparing the viability and proliferation of NHDFs following 7 mW/cm^2^ irradiation, we found that the corresponding peak viability at 5.04 J/cm^2^ was not related to the peak proliferation at 1.05 J/cm^2^. This discrepancy could be attributed to the differences between the two assays. Assays of cell proliferation measured the amount of newly synthesized DNA within the time frame of 24 h after irradiation. Assays of cell viability relied on transient metabolic activities 24 h after irradiation.

In addition to keratinocytes and fibroblasts, endothelial cells also play crucial roles in the process of wound healing. So far, studies on BL irradiation are limited. Rohringer et al. reported a trend toward increased proliferation at 72 h after 24 J/cm^2^ irradiation in HUVECs [43]. Dungel et al. reported that 30 J/cm^2^ BL irradiation in a rat skin flap model had proangiogenic effects [44]. In addition, more research has focused on the red light spectrum. Based on previous studies [30] on HUVECs after 453 nm pulsed BL, the HUVECs in our study provided some updates: the continuous mode of BL irradiation can promote the viability, proliferation, and migration of HUVECs as well, irrespective of irradiance of 7, 10, or 23 mW/cm^2^. These findings provide an experimental basis for PBM for angiogenesis after continuous irradiation.

Multiple factors, including cell culture conditions (serum concentration, confluence, and environmental oxygen levels) and treatment protocols (medium refreshment and medium irradiation), potentially influenced cellular responses after irradiation [45]. Additionally, varying irradiation modes with various wavelengths and fluences, along with known cell type-dependent behaviors, make it even more difficult to directly compare conclusions on PBM. In our study, medium replenishment was not performed after irradiation to induce a simultaneous effect from the microenvironment. Riboflavin, as a medium component, has been shown to act as a photosensitizer to induce the production of ROS, which partly accounts for the discrepancy in conclusions compared with other PBM-related studies [46]. Medium irradiation followed by transferring the irradiated medium to non-irradiated cells allowed photon interactions with medium components and generated secondary products (Figure S16). The findings are aligned with the conclusions reported by Almeida-Lopes et al, who observed that serum concentration influenced the growth of gingival fibroblasts with a fluence at 2 J/cm^2^ [47]. These findings suggest that medium composition (e.g., serum and riboflavin) can influence the process of PBM. However, as an alternative solution to avoid medium interference during irradiation, DPBS is not selected in our study, because high-fluence irradiation is also investigated. Prolonged incubation in DPBS can cause irreversible damage due to nutritional deficit, introducing a confounding factor that complicates the interpretation of inhibitory effects after BL irradiation. In contrast, medium provides a more physiologically relevant microenvironment, better simulating in vivo conditions and facilitating the application in animal models and clinical practices.

Based on the biological effects of 4.5 and 18 J/cm^2^ irradiation measured in different assays, RNA sequencing was performed to explore the upregulated and downregulated genes and the enriched pathways related to PBM. After enrichment analysis, 4.5 J/cm^2^ irradiation was found to upregulate the ErbB, TGF-β, and VEGF pathways. The ErbB pathway is related to the epidermal growth factor receptor (EGFR) family. Jere et al. reported the PBM of fibroblasts by the expression of epidermal growth factor (EGF) after exposure to 5 J/cm^2^ 660 nm irradiation, indicating that PBM activated the JAK/STAT pathway and promoted proliferation and migration [48]. Karoussis et al. reported that a 12 J/cm^2^ 810 nm laser promoted gingival fibroblast proliferation through expression of the EGF gene [49]. Although there were some differences in the light source parameters, the activation of PBM via EGF after low-dose light treatment has been proven. The enrichment analysis revealed that 450 nm BL irradiation upregulated the ErbB pathway, and the downstream responses of cell viability and proliferation were subsequently promoted. Through differential expression analysis, significant upregulation of epiregulin (EREG) was detected, suggesting that cellular proliferation was likely promoted through interaction with EGFR. We also detected the upregulation of MEMO1, which was originally identified as a molecule associated with the ErbB2 receptor and was determined to be essential for effective cell migration [50]. The TGF-β pathway can trigger versatile processes contributing to wound healing, such as fibroblast activation, angiogenesis, and re-epithelialization. The activation of the PI3K/AKT and JAK/STAT signaling pathways by TGF-β is related to multiple cellular responses, including metabolism, growth, and proliferation [51]. With respect to BL, Mignon et al. applied a dose of 30 J/cm^2^ to two subpopulations of fibroblasts and reported that BL treatment induced inhibitory effects through downregulating the TGF-β pathway [37]. The upregulated genes in the VEGF pathway contribute to the process of angiogenesis. This increased expression of genes in this pathway stimulates the formation of vessels, which has been widely reported [52,53,54]. In our study, the enrichment of the VEGF pathway indicated that activated fibroblasts were involved in angiogenesis after 450 nm BL irradiation. We also found that 4.5 J/cm^2^ irradiation induced significant upregulation of MET, and bioinformatic analysis of the PPI network indicated its pivotal role in PBM. HGF/c-MET signaling has been shown to be involved in cutaneous wound healing [55,56]. The upregulated expression of c-MET in mature tissue promoted neovascularization and granulation tissue formation [57]. The significantly high expression of COL5A3 and COL8A1 stimulated the biosynthesis of collagen after 4.5 J/cm^2^ BL irradiation. Collagen V, a type of fibrillar collagen, ensures structural integrity and tissue support. It also modulates cellular behaviors and functions [58]. As a nonfibrillar and short-chain collagen, collagen VIII is involved in vascular remodeling by regulating the migration, proliferation, and adhesion of vascular smooth muscle cells [59]. These increased expressions of collagen-related genes indicate their facilitation in skin regeneration.

In contrast, the results of RNA sequencing revealed that 18 J/cm^2^ BL irradiation influenced primarily the processes of DNA replication, the cell cycle, and mismatch repair in fibroblasts, with the downregulation of some mitosis-related genes and genes regulating the cell cycle. Combined with the results of the apoptosis assays, our findings indicated that 18 J/cm^2^ BL irradiation had inhibitory effects on proliferation and the cell cycle without inducing apoptosis or cell death. However, from the PPI network of 18 J/cm^2^, TP53 was found to have the most connections with other differentially expressed genes, indicating that the inhibitory effects on DNA replication, the cell cycle, and mismatch repair have wide connections with this apoptosis-related gene.

Notably, no statistically significant anti-apoptotic or pro-apoptotic effects were observed following 4.5 J/cm^2^ BL irradiation, as indicated by comparisons of cell proportions in quadrants of early/late apoptosis to their respective controls (Figure S11). Our findings are in line with a prior study by Trajano et al. [60], who reported 10 J/cm^2^ low-level infrared laser (880 nm) did not alter the apoptotic cell populations of C2C12 myoblast cells 24, 48, or 72 h post-irradiation. Jiang et al. also reported consistent results after 6 and 30 J/cm^2^ BL irradiation (457 nm) on oral squamous cell carcinoma cell lines, without an increase in percentage of apoptosis [61]. Therefore, the absence of a significant apoptotic change in our study aligns with previous findings that BL exerts neither anti-apoptotic nor cytotoxic effects under the condition of low-fluence irradiation.

Our findings regarding PBM were obviously inconsistent with what was concluded in photoaging. Photoaging is a multifactorial and complex process after light exposure. Light treatment raises matrix metalloproteinase (MMP) levels by interaction with photoreceptors, followed by the production of ROS and DNA damage caused by light [62]. Avola et al. verified that both 15 and 45 J/cm^2^ BL triggered oxidative stress in keratinocytes and further led to premature aging [63]. Ge et al. found significantly downregulated type I collagen genes and upregulated MMP1 after adding 10, 15, and 20 J/cm^2^ irradiation on fibroblasts [64]. These contradictory conclusions regarding keratinocytes and fibroblasts are probably due to differences in light doses. As research on photoaging primarily focuses on the adverse effects of light exposure, with radiation doses generally higher than those used in PBM studies. A review of PBM effects on keratinocytes found that cells benefited from doses between 0.1 and 5 J/cm^2^ [65]. In terms of BL irradiation on fibroblasts, Prado et al. listed energy densities among studies of PBM on fibroblasts, indicating that an energy density of <5 J/cm^2^ yields favorable results on the healing process [66].

According to the Beer–Lambert law and Planck’s law, the wavelength of visible light is directly proportional to penetration depth and inversely proportional to energy [67,68]. Therefore, utilizing BL irradiation on superficial tissue becomes one of the advantages. Combining with its anti-inflammatory property and PBM at low fluence, BL has broad clinical perspectives as a therapeutic approach, especially in the treatment of skin lesions. Cai et al. reported that BL at a fluence of 4.8 J/cm^2^ promoted wound closure rates in diabetic rat models, accompanied by better anti-inflammatory effects than red light [69]. The expression levels of IL-6 and TNF-α were both lower in the BL group compared with red light irradiation. Dini et al. applied BL irradiation with a wavelength between 400 and 430 nm on patients afflicted by chronic ulcers and achieved satisfactory results in wound healing and pain relief [70]. Spinella et al. conducted a retrospective study focusing on BL treatment following skin ulcers related to systemic sclerosis [71]. Both groups reported a clear reduction in ulcer area and pain under BL. It remains to be shown if BL irradiation at 450 nm can also clinically improve the prognosis of ulcers with other etiologies at low fluence. 

Our study indicates the cell type- and dose-dependent effects of BL on HaCaTs, NHDFs, and HUVECs. Transcriptomic and bioinformatic analyses revealed pathways associated with both stimulatory and inhibitory responses following BL exposure. While these findings enhance our understanding of wound healing and angiogenesis, particularly in response to 450 nm BL, and provide foundational evidence for potential future clinical applications, this study also presents several limitations. Notably, cell type-specific responses must be considered when translating PBM into clinical practice. We used immortalized keratinocytes which may show a different response than primary keratinocytes. A more comprehensive conclusion would involve a direct comparison of the responses between HaCaTs and primary keratinocytes under BL conditions. Additionally, interactions between BL and medium components induce secondary effects that significantly influence cellular responses post-irradiation. These effects contributed to increased cell viability, most prominently in HUVECs (84%), followed by NHDFs (57%) and HaCaTs (44%). Nonetheless, eliminating such confounding factors remains difficult, as the culture medium is essential for maintaining cell viability and function during extended irradiation. Here controls using DPBS under irradiation would impair cell viability. Moreover, performing irradiation within cells’ native microenvironments, i.e., an in vivo model for wound healing, would offer a more physiologically relevant model. Importantly, the potential adverse effects of BL—such as hyperpigmentation and carcinogenesis—necessitate caution in future clinical application [72,73]. Finally, further mechanistic investigations, particularly using transcriptomic analysis of keratinocytes and endothelial cells, will be crucial for future studies to elucidate the interactions between these cell types during wound healing and angiogenesis.

## 5. Conclusions

BL irradiation at a wavelength of 450 nm induced biphasic cellular responses in HaCaTs, NHDFs, and HUVECs, with cell type-dependent stimulatory effects on cell viability, proliferation, migration, and ATP synthesis at a fluence of 4.5 J/cm^2^. Conversely, 18 J/cm^2^ BL had the opposite effect on the three cell types, inhibiting cell viability and proliferation. Exposure to 4.5 J/cm^2^ BL irradiation activated NHDFs via the ErbB, TGF-β, and VEGF signaling pathways, whereas 18 J/cm^2^ irradiation inhibited DNA replication, cell cycle, and mismatch repair pathways.

## Data Availability

The original contributions presented in this study are included in the article/Supplementary Material. Further inquiries can be directed to the corresponding author(s).

## Supplementary Materials

The following supporting information can be downloaded at https://www.mdpi.com/article/10.3390/biomedicines13081876/s1. Figure S1: Morphologies of HUVECs, Figure S2: Osram Duris S5, GD PSLR31, Figure S3: Spectral Emission, Figure S4: Temperature Measurements, Figure S5: REUR2021-085SAF Photodiode-Based Device and a Metal Plate, Figure S6: Heatmap for Light Homogeneity (Percentage), Figure S7: HaCaT Migration Assays; Figure S8: NHDF Migration Assays, Figure S9: HUVEC Migration Assays, Figure S10: Apoptosis, Figure S11: Apoptosis by quadrant, Figure S12: CorHeatmap, Figure S13: SummaryPlot, Figure S14: Reactome_GSEA, Figure S15: PPI Network High Dose, Figure S16: Medium Irradiation Followed By Transferring The Irradiated Medium To Non-Irradiated Cells, Table S1: Irradiation Parameter With Fluences.
