# Supplementary material for: Photobiomodulation of 450 nm Blue Light on Human Keratinocytes, Fibroblasts, and Endothelial Cells: An In Vitro and Transcriptomic Study on Cells Involved in Wound Healing and Angiogenesis
Source: Biomedicines. 2025 Aug 1;13(8):1876. doi: 10.3390/biomedicines13081876 (PMC12383942; doi:10.3390/biomedicines13081876)
Supplement: Supplementary file 1 [file biomedicines-13-01876-s001.zip › Supplementary/Table S1. Irradiation Parameters With Fluence.pdf]

Table S1. Irradiation time used to achieve specific fluences under each irradiance

Irradiance	7 mW/cm²	10 mW/cm²	23 mW/cm²
	Fluence (J/cm²)		
2 min 30 s	1.05	1.5	3.45
5 min	2.1	3	6.9
7 min 30 s	3.15	4.5	10.35
10 min	4.2	6	13.8
12 min	5.04	7.2	16.56
15 min	6.3	9	20.7
20 min	8.4	12	27.6
30 min	12.6	18	41.4
60 min	25.2	36	82.8
90 min	37.8	54	124.2
120 min	50.4	72	165.6

Note: Irradiation time is expressed in minutes (min) and seconds (s).
