# Supplementary figures and images for: Photobiomodulation of 450 nm Blue Light on Human Keratinocytes, Fibroblasts, and Endothelial Cells: An In Vitro and Transcriptomic Study on Cells Involved in Wound Healing and Angiogenesis
Source: Biomedicines. 2025 Aug 1;13(8):1876. doi: 10.3390/biomedicines13081876 (PMC12383942; doi:10.3390/biomedicines13081876)

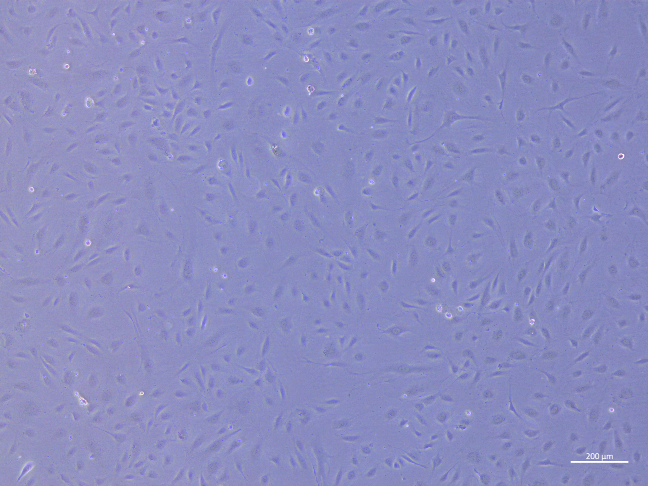

Supplement: Supplementary file 1 [file biomedicines-13-01876-s001.zip › Supplementary/Figure S1 Morphologies of HUVECs.tiff]

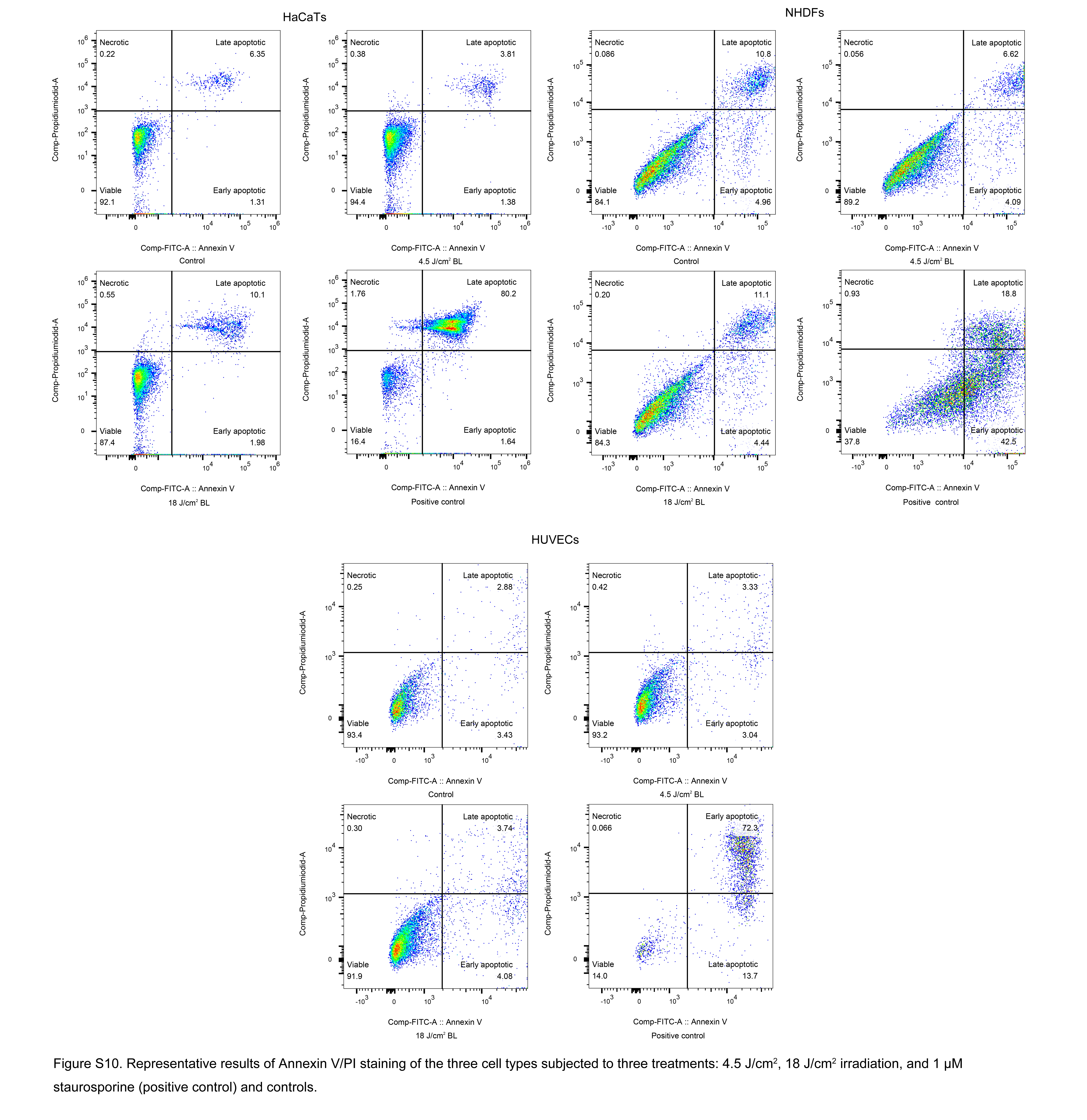

Supplement: Supplementary file 1 [file biomedicines-13-01876-s001.zip › Supplementary/Figure S10. Apoptosis assay.tif]

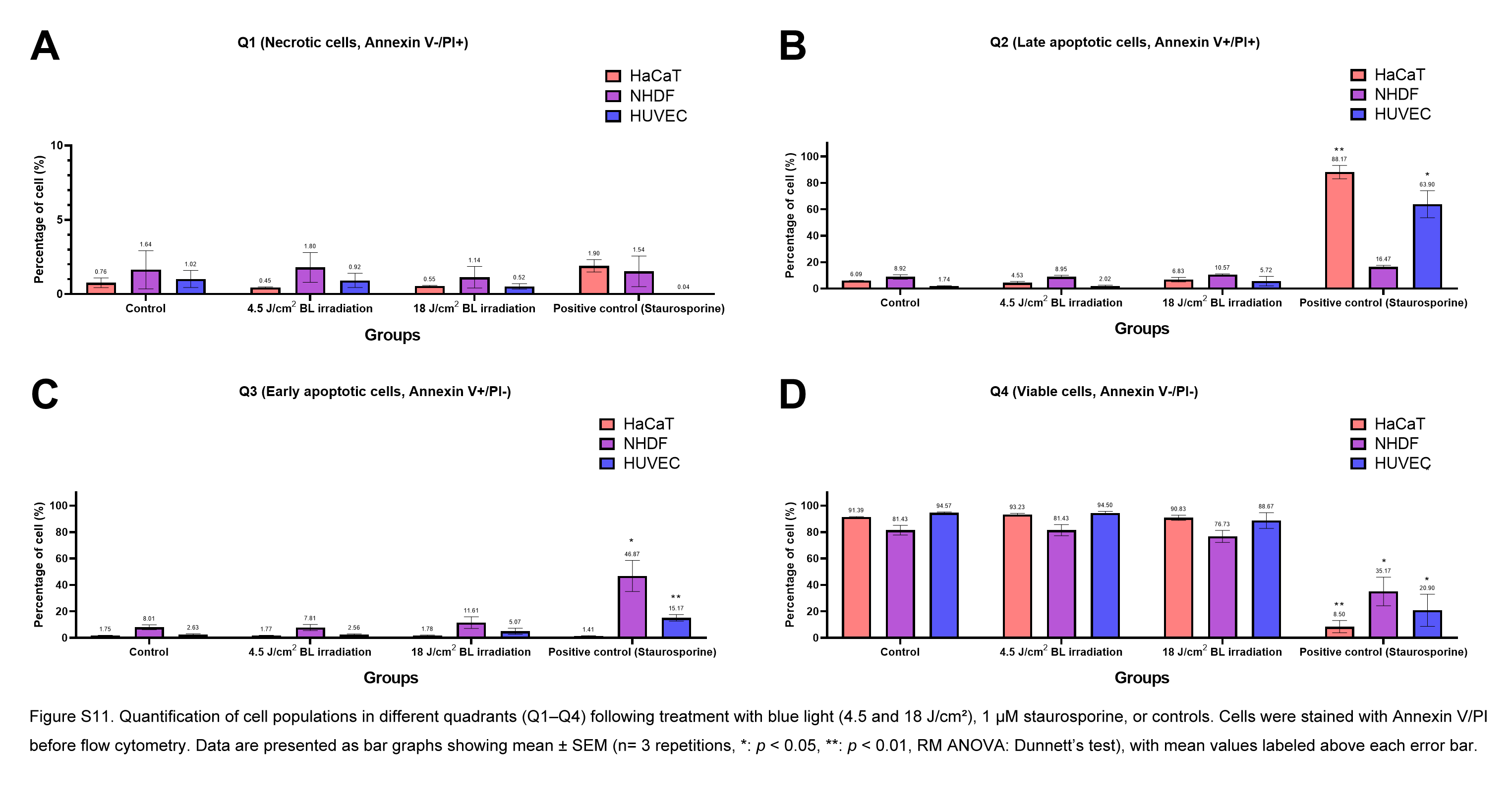

Supplement: Supplementary file 1 [file biomedicines-13-01876-s001.zip › Supplementary/Figure S11. Apoptosis by quadrant.tif]

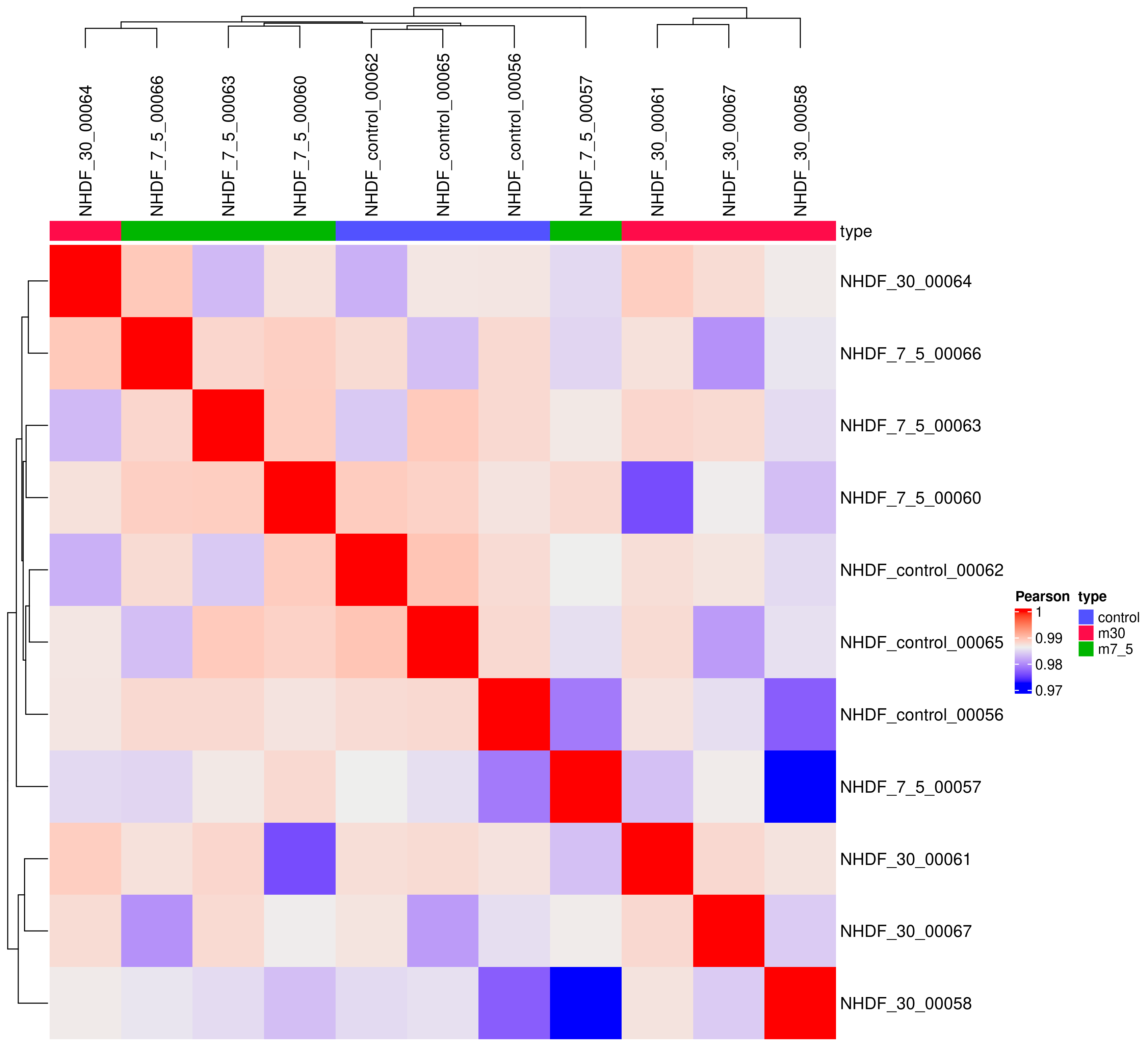

Supplement: Supplementary file 1 [file biomedicines-13-01876-s001.zip › Supplementary/Figure S12.CorHeatmap.png]

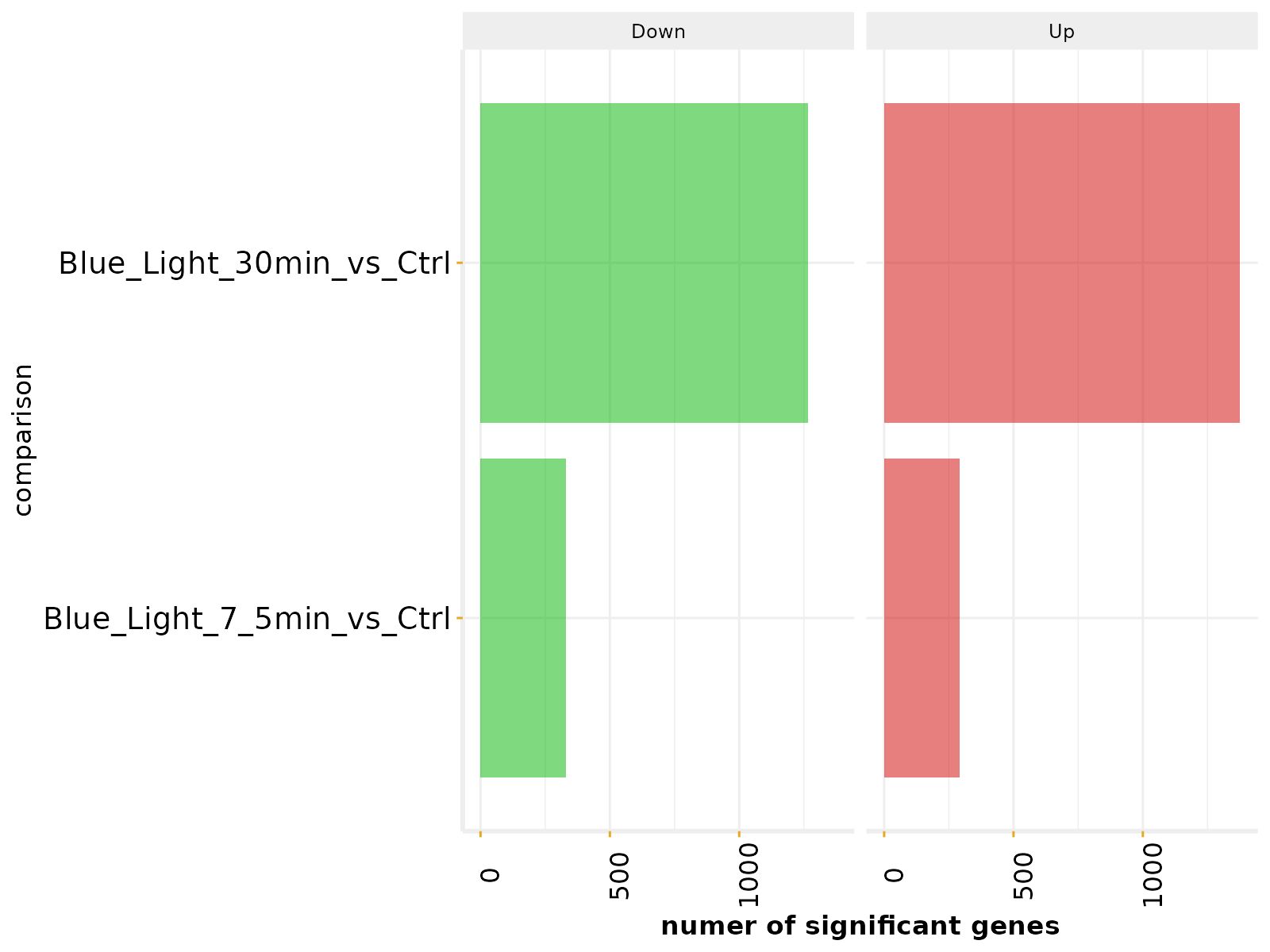

Supplement: Supplementary file 1 [file biomedicines-13-01876-s001.zip › Supplementary/Figure S13. SummaryPlot.png]

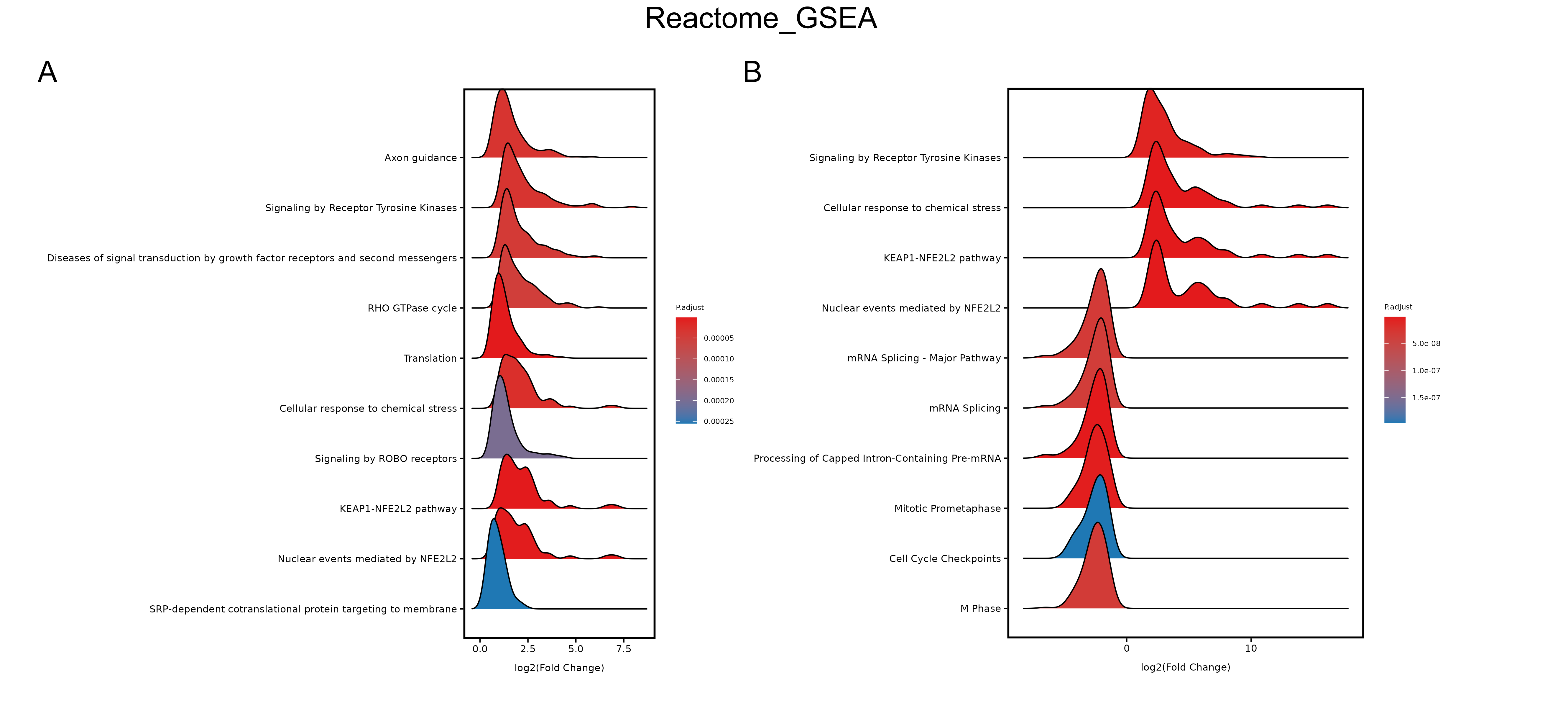

Supplement: Supplementary file 1 [file biomedicines-13-01876-s001.zip › Supplementary/Figure S14. Reactome_ GSEA.tif]

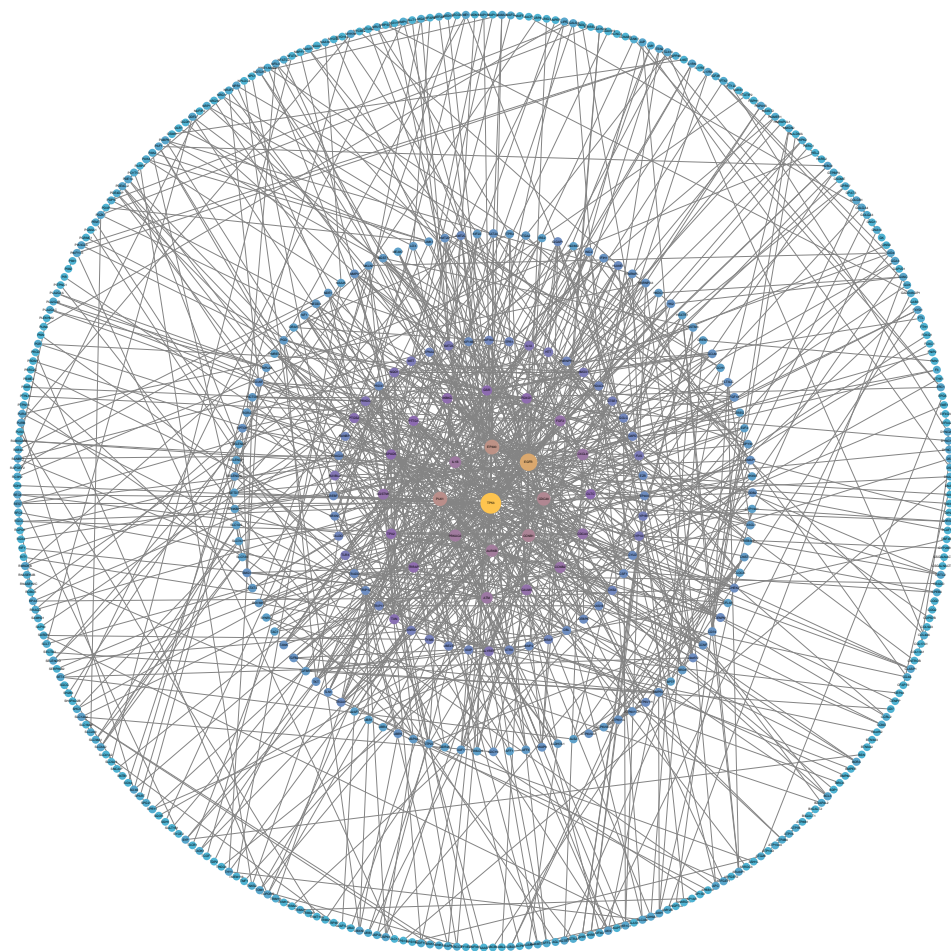

Supplement: Supplementary file 1 [file biomedicines-13-01876-s001.zip › Supplementary/Figure S15. PPI network high dose.pdf]

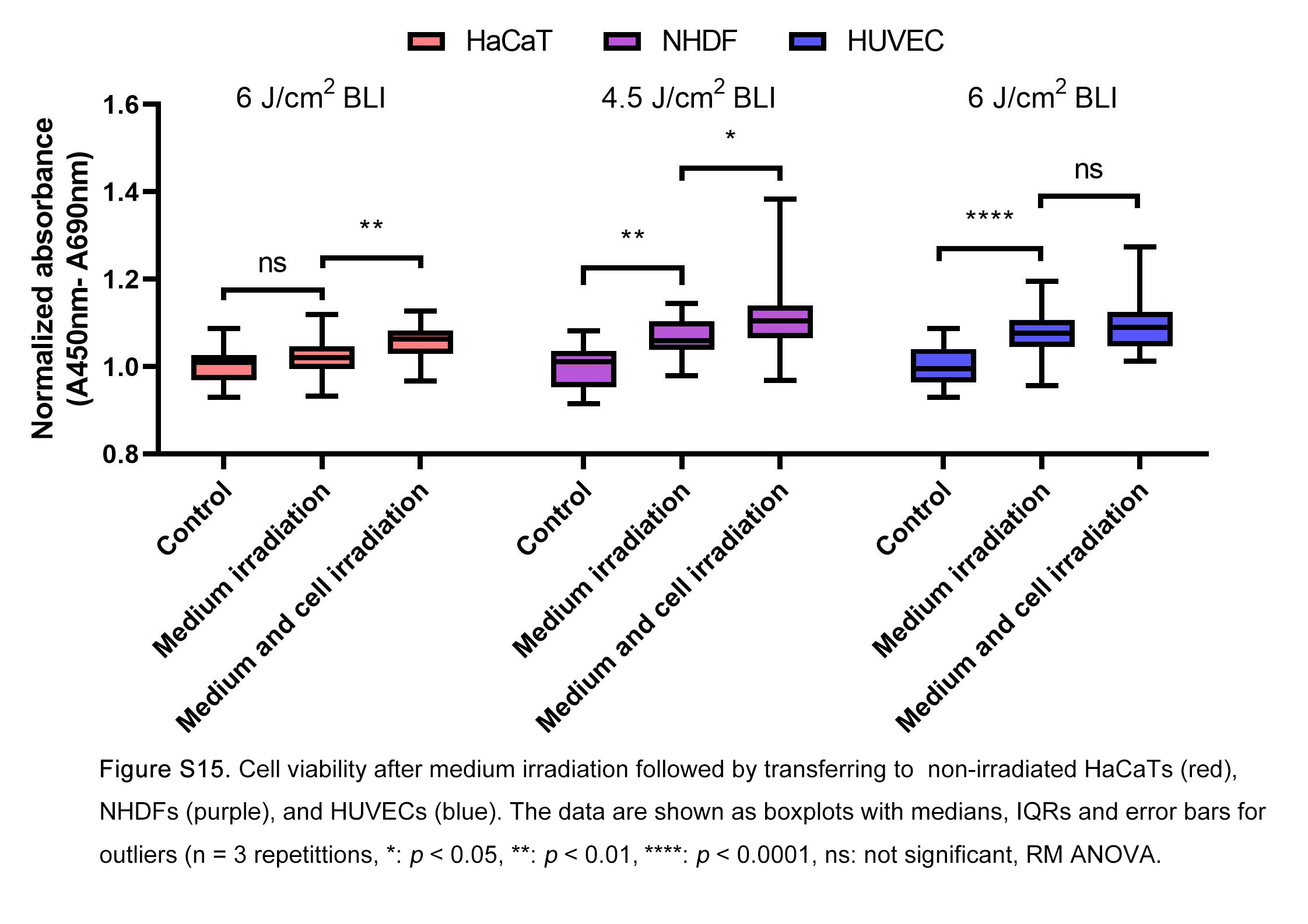

Supplement: Supplementary file 1 [file biomedicines-13-01876-s001.zip › Supplementary/Figure S16. Medium Irradiation Followed By Transferring The Irradiated Medium to Non-irradiated Cells.jpg]

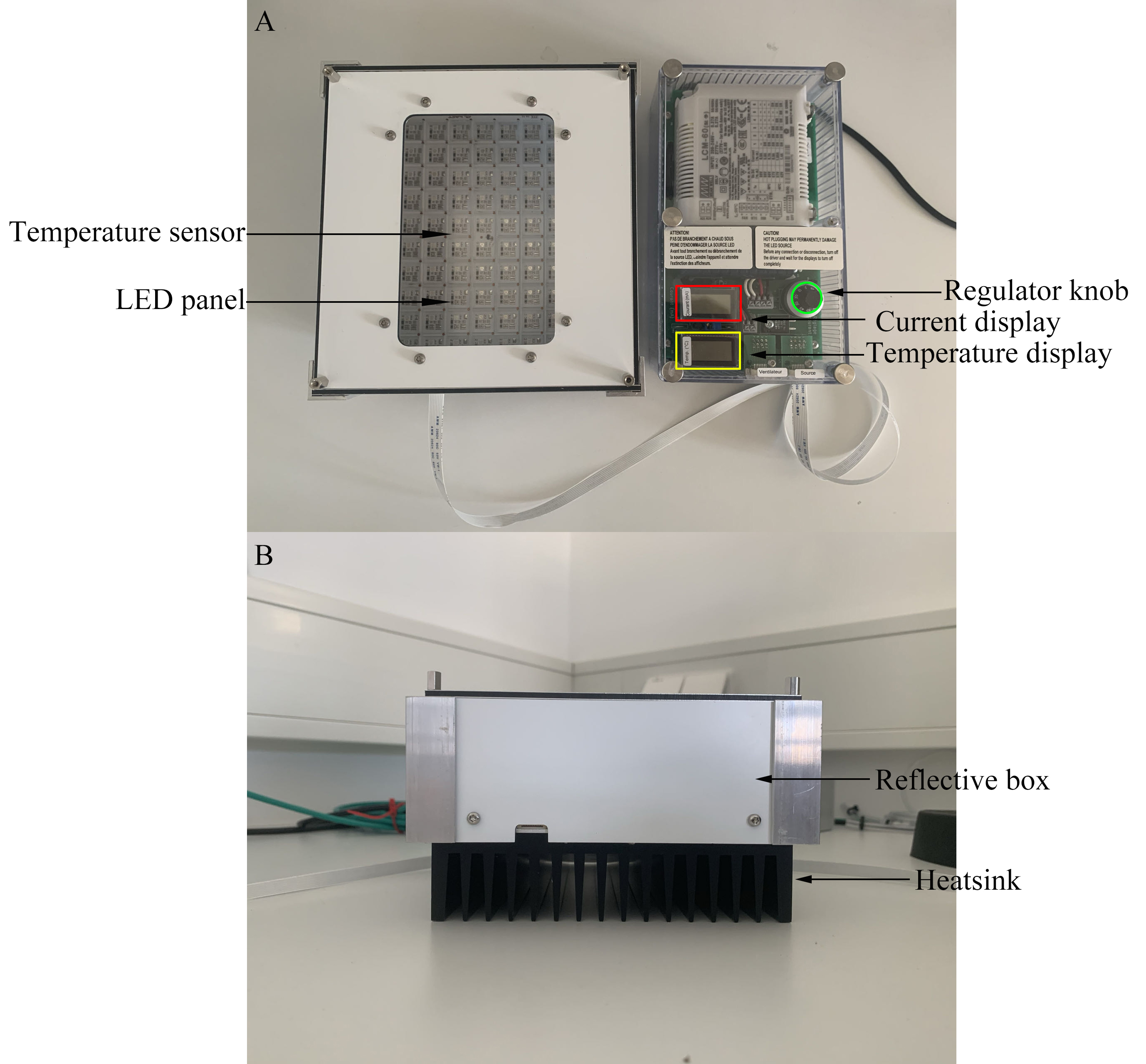

Supplement: Supplementary file 1 [file biomedicines-13-01876-s001.zip › Supplementary/Figure S2. Osram Duris S5, GD PSLR31.jpg]

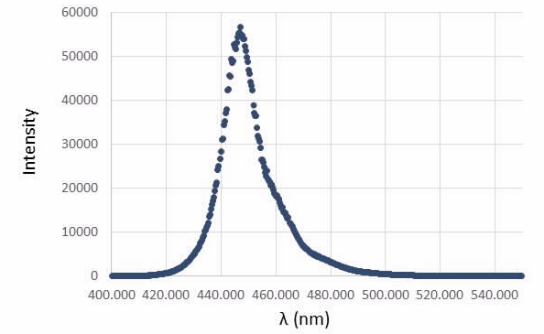

Supplement: Supplementary file 1 [file biomedicines-13-01876-s001.zip › Supplementary/Figure S3. Spectral Emission.tif]

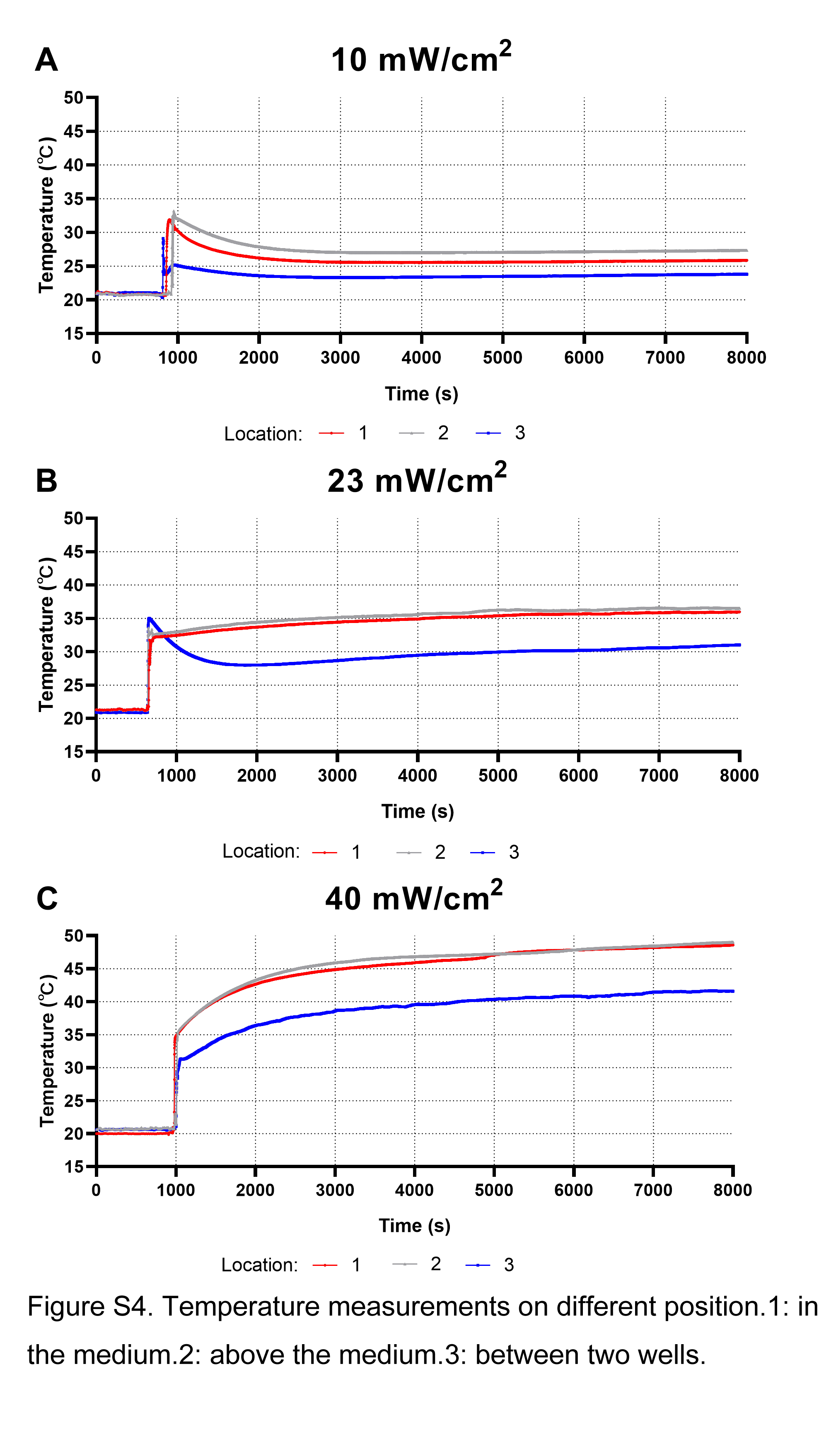

Supplement: Supplementary file 1 [file biomedicines-13-01876-s001.zip › Supplementary/Figure S4. Temperature Measurements.tif]

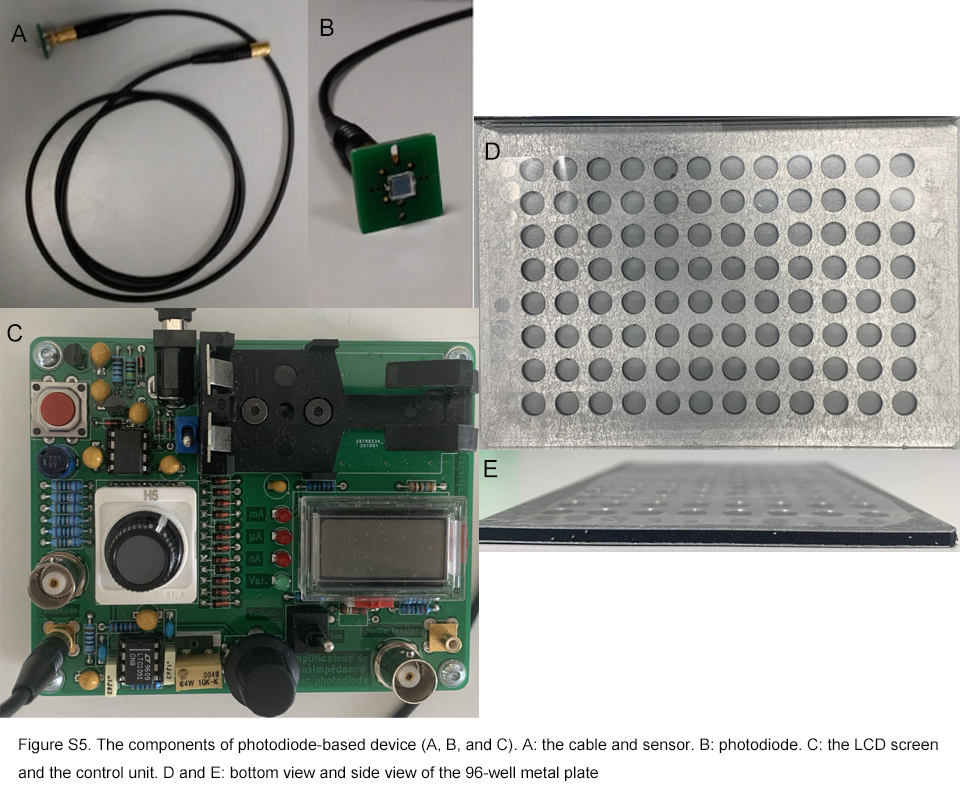

Supplement: Supplementary file 1 [file biomedicines-13-01876-s001.zip › Supplementary/Figure S5. REUR2021-085SAF Photodiode-Based Device and a Metal Plate.tif]

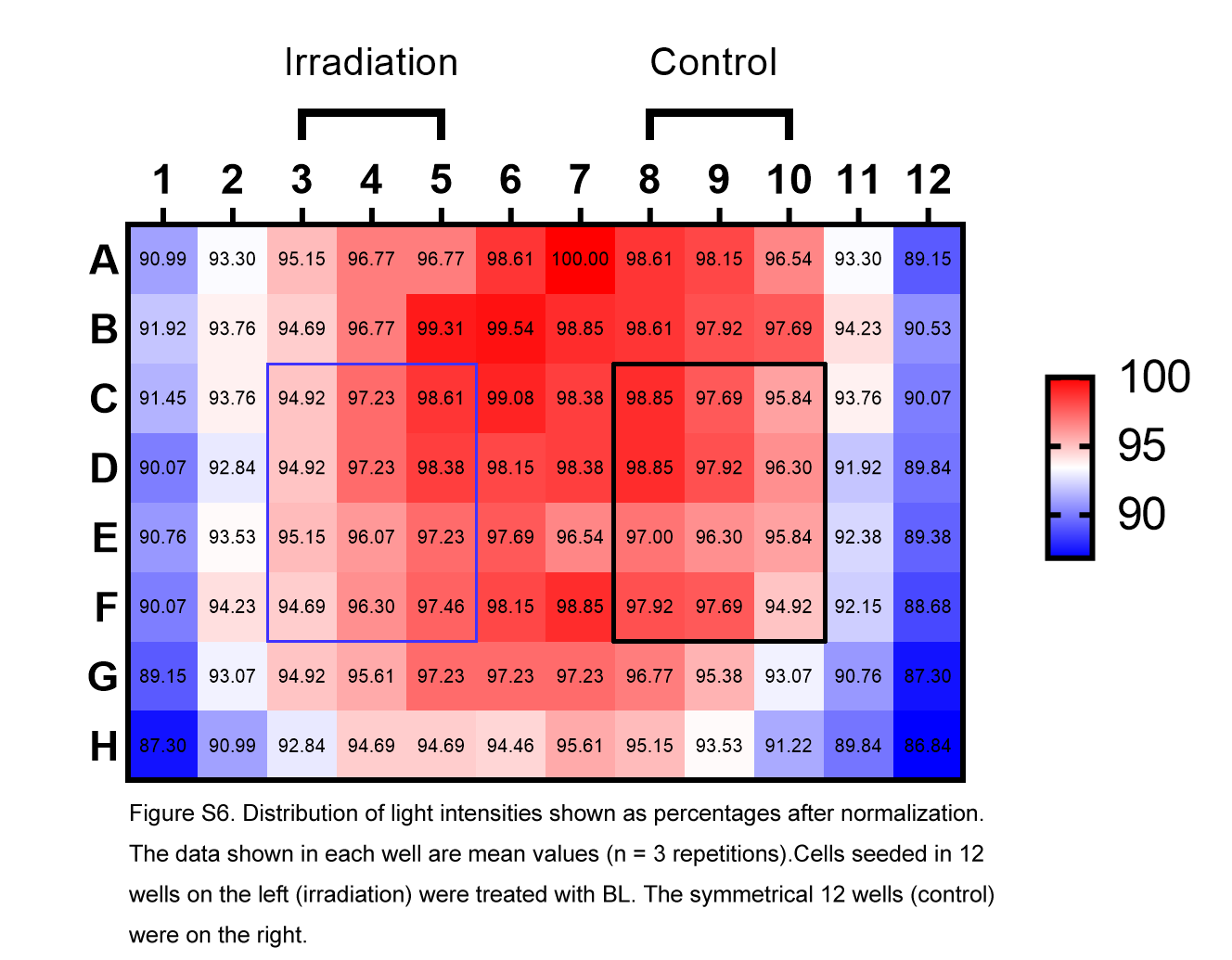

Supplement: Supplementary file 1 [file biomedicines-13-01876-s001.zip › Supplementary/Figure S6. Heatmap for Light Homogeneity (Percentage).tif]

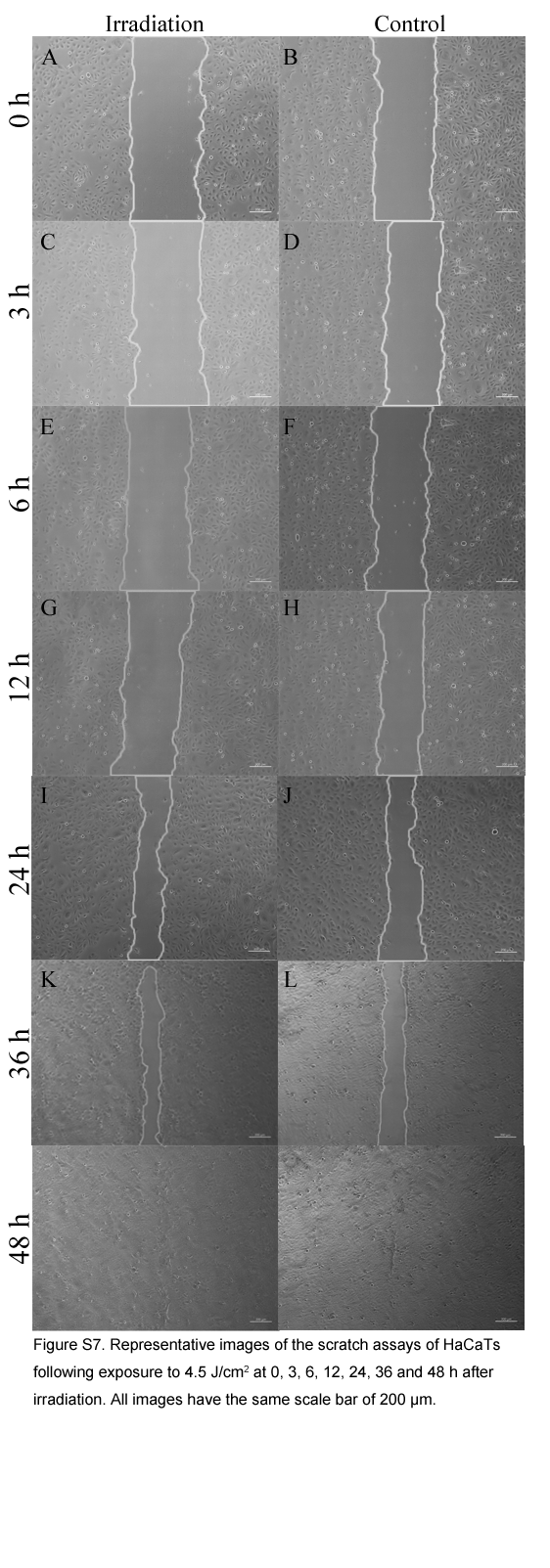

Supplement: Supplementary file 1 [file biomedicines-13-01876-s001.zip › Supplementary/Figure S7.HaCaT migration assays.tif]

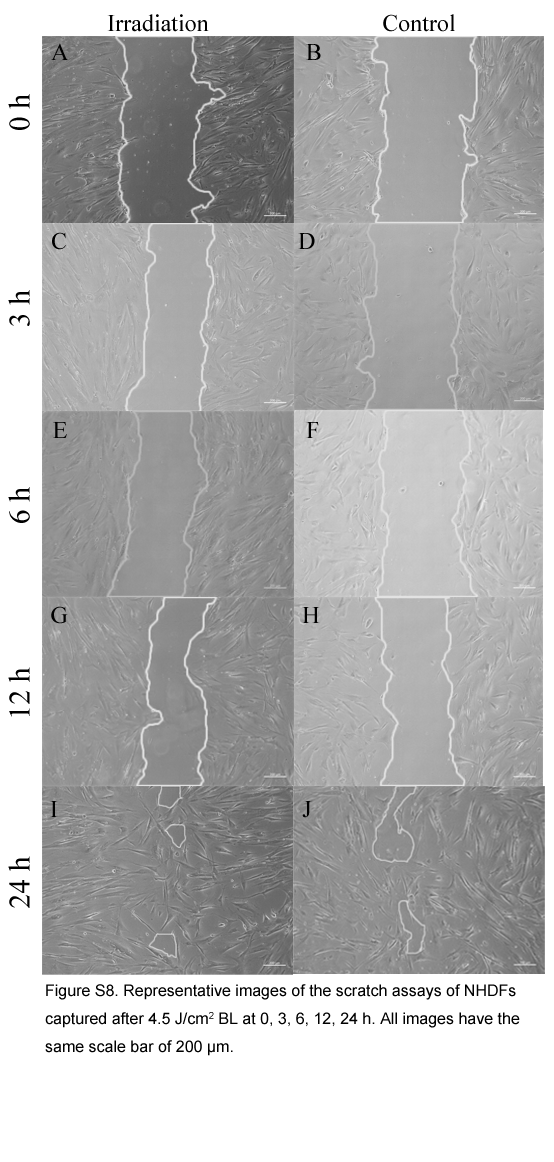

Supplement: Supplementary file 1 [file biomedicines-13-01876-s001.zip › Supplementary/Figure S8. NHDF migration assays.tif]

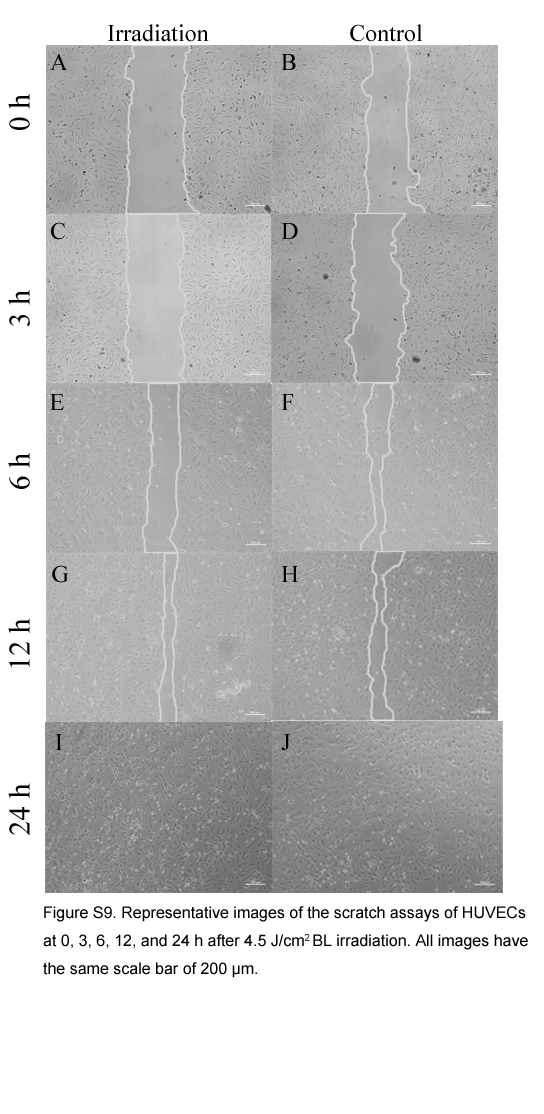

Supplement: Supplementary file 1 [file biomedicines-13-01876-s001.zip › Supplementary/Figure S9. HUVEC migration assays.tif]
